# Schizophrenia-associated Mitotic Arrest Deficient-1 (MAD1) regulates the polarity of migrating neurons in the developing neocortex

**DOI:** 10.1038/s41380-022-01856-5

**Published:** 2022-11-10

**Authors:** Bon Seong Goo, Dong Jin Mun, Seunghyun Kim, Truong Thi My Nhung, Su Been Lee, Youngsik Woo, Soo Jeong Kim, Bo Kyoung Suh, Sung Jin Park, Hee-Eun Lee, Kunyou Park, Hyunsoo Jang, Jong-Cheol Rah, Ki-Jun Yoon, Seung Tae Baek, Seung-Yeol Park, Sang Ki Park

**Affiliations:** 1 Department of Life Sciences, Pohang University of Science and Technology, Pohang, 37673 Republic of Korea; 2 Department of Biological Sciences, Korea Advanced Institute of Science and Technology (KAIST), Daejeon, 34141 Republic of Korea; 3 Korea Brain Research Institute, Daegu, 41062 Republic of Korea; 4 Present Address: Program in Molecular Medicine, University of Massachusetts Medical School, Worcester, MA 01655 USA

**Keywords:** Neuroscience, Cell biology

## Abstract

Although large-scale genome-wide association studies (GWAS) have identified an association between *MAD1L1* (*Mitotic Arrest Deficient-1 Like 1*) and the pathology of schizophrenia, the molecular mechanisms underlying this association remain unclear. In the present study, we aimed to address these mechanisms by examining the role of MAD1 (the gene product of *MAD1L1)* in key neurodevelopmental processes in mice and human organoids. Our findings indicated that MAD1 is highly expressed during active cortical development and that MAD1 deficiency leads to impairments in neuronal migration and neurite outgrowth. We also observed that MAD1 is localized to the Golgi apparatus and regulates vesicular trafficking from the Golgi apparatus to the plasma membrane, which is required for the growth and polarity of migrating neurons. In this process, MAD1 physically interacts and collaborates with the kinesin-like protein KIFC3 (kinesin family member C3) to regulate the morphology of the Golgi apparatus and neuronal polarity, thereby ensuring proper neuronal migration and differentiation. Consequently, our findings indicate that MAD1 is an essential regulator of neuronal development and that alterations in MAD1 may underlie schizophrenia pathobiology.

## Introduction

Schizophrenia (SZ) is a major psychiatric disorder that affects many aspects of behavior and cognition [1, 2]. According to the neurodevelopmental hypothesis of SZ pathogenesis, anomalies in neuronal connectivity that occur during various stages of neurodevelopment predispose individuals to SZ [3]. Patients with SZ exhibit anatomical changes in the brain, such as reduced cortical volume, enlarged ventricles, and diminished cortical gray matter [4]. Such changes have been attributed to defective neurodevelopmental processes, including proliferation, migration, differentiation, and synaptogenesis [5].

Several human studies, including twin studies and genome-wide association studies (GWAS), have indicated that the genetic contribution to SZ may be up to 80% [3, 6]. Some large-scale GWAS have identified novel genetic loci associated with SZ, including *MAD1L1* [7–10]. DNA methylation profiling studies of human patients with SZ also suggested *MAD1L1* as a novel risk locus [11, 12]. Moreover, protein interactome networks of SZ-associated factors place MAD1 as one of the interaction hubs harboring 53 protein-protein interactions [13]. These findings collectively indicate that *MAD1L1* is a potentially critical factor associated with SZ pathobiology.

MAD1 regulates the spindle assembly checkpoint (SAC) during mitosis [14]. During metaphase, MAD1 is localized to the kinetochore and recruits MAD2 to further the mitotic checkpoint complex (MCC) [15, 16]. When the spindle is not adequately attached to the kinetochore, the MCC mediates mitotic arrest by inhibiting chromosome segregation [17]. MAD1 also ensures linkage between kinetochores and microtubules via centromere-associated protein E (CENP-E), a microtubule plus-end-directed kinesin motor that mediates chromosome congression [18]. Mutation or alteration of *MAD1L1* expression increases the risks of cancer [19, 20] and abnormal mitosis (e.g., aneuploidy) [21]. The MAD1 fraction in the cytoplasm localizes to the Golgi apparatus and regulates the secretion of α5-integrin for proper cell migration and adhesion [22]. Although the functions of MAD1 during cell division are relatively well established, its roles in other biological processes are largely unexplored.

The Golgi apparatus plays a pivotal role in determining cellular polarity, secretory pathways, and posttranslational modifications of secreted proteins. The Golgi apparatus is positioned in the proximity of the centrosome to assign the direction of cellular polarity, which enables changes in cell shape, polarized cargo trafficking, and unidirectional migration [23]. The centrosome–Golgi axis directs intracellular cargo trafficking for proper neuronal morphogenesis and functionality [24, 25]. During neurodevelopment, it determines the fidelity of neuronal migration and protrusion sites of differentiating neurons [23, 26]. Therefore, the configuration of the centrosome–Golgi axis in neuronal cells is thought to be one of the critical regulatory points in the progression of neurodevelopment.

In the present study, we aimed to investigate the regulatory role of MAD1 in neurodevelopmental processes. Our findings indicated that MAD1 regulates neuronal migration and differentiation by determining the polarity of migrating neurons and that the kinesin-like protein KIFC3 (kinesin family member C3) is a crucial mediator of MAD1 function due to its role in regulating Golgi morphology and membrane trafficking.

## Materials and methods

### Animals

For mouse embryos, pregnant C57BL/6 mice were purchased from Hyochang Science (Daegu, South Korea). E15 or E16 embryos were used for primary culture. For in utero electroporation, E13.5 or E14.5 embryos were used. All animal experiments were ethically approved by the Institutional Animal Care and Use Committee (IACUC) of Pohang University of Science and Technology (POSTECH-2019-0024, POSTECH-2019-0025, POSTECH-2020-0018, and POSTECH-2020-0022).

### Statistical analysis

The sample size was determined by non-statistical methods based on previous studies which used experimental approaches and result analyses [22, 23, 26–33]. We also evaluated our experimental design based on statistical theories well accepted in the field; Sample number above 30 is recommended to meet the central limit theorem and the minimum number of independent sets is 3 to analyze the experimental results statistically [34]. We tried to meet the minimum cell counting number above 30 cells. Sample sizes of the animal experiments and set compositions were designed following the previous studies (at least 3 mice per experimental group) [35] and ethical guidelines. No randomization was conducted in animal studies. No blinding was done for the group allocation, including the animal experiments. For statistical analysis, GraphPad Prism 9.0 (GraphPad Software) was used. Unpaired two-tailed Student’s *t*-test was used for comparison between two groups, and one-way ANOVA followed by Turkey’s post-hoc test was used for comparison of multiple groups. Additional analysis with nested *t*-test and nested one-way ANOVA was performed (Supplementary dataset). Two-way ANOVA followed by Turkey’s post-hoc test was used for Sholl analysis. All graphs were presented as mean ± SEM.

Materials and methods are further described in the Supplementary Methods.

## Results

### MAD1 regulates neuronal migration in the developing neocortex

To examine the potential participation of MAD1 in the nervous system, we first assessed *MAD1L1* expression in the mouse brain. Gene expression databases such as Bgee (https://bgee.org) and Expression Atlas (https://www.ebi.ac.uk/gxa/home) indicate significant expression of *MAD1L1* in the early forebrain of mice and humans. We collected whole brains from C57BL/6 mice from embryonic day 10 (E10) to E18, at postnatal day 1 (P1), and during adulthood, following which we performed semi-quantitative RT-PCR analysis against the *MAD1L1* gene. *MAD1L1* expression was significantly higher in early developing brains, with a peak at E16 (Supplementary Fig. 1a). Consistently, we also detected significant expression of MAD1 in the embryonic cortex and hippocampus (Supplementary Fig. 1b). Additionally, in situ hybridization database Allen Brain Atlas (https://portal.brain-map.org) suggests that *MAD1L1* gene is widely expressed in mouse and human brain, including the cortex and hippocampus with strong expression in the developing brain. The single-cell RNA-seq database (https://portal.brain-map.org) also demonstrated the significant expression *MAD1L1* gene in various neuronal cell populations, including neural stem cells, progenitor cells, and differentiating and mature neurons [36]. Therefore, our observations and previous analysis consistently suggest that *MAD1L1* is widely expressed in the nervous system, with a higher expression at the developing stages.

**Fig. 1:**
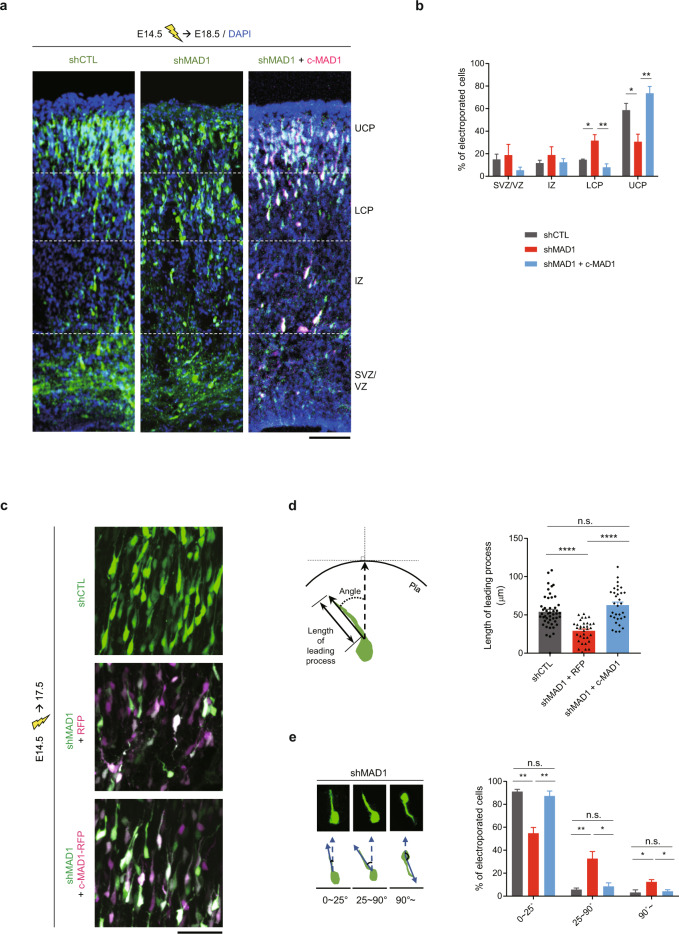
MAD1-deficiency leads to delayed neuronal migration in the developing cortex. **a**, **b** C57BL/6 mouse embryos were electroporated with scrambled control shRNA (shCTL), MAD1 shRNA (shMAD1), or shMAD1 with RFP-tagged c-MAD1 at E14.5 and analyzed at E18.5. Transfected cells are GFP-positive for shCTL or shMAD1 and RFP-positive (magenta) for c-MAD1. Representative images (**a**) and statistical analysis of neuronal migration (**b**, shCTL, *n* = 3 brains; shMAD1, *n* = 3 brains; shMAD1 + c-MAD1, *n* = 3 brains). **c**–**e** Mouse embryos were electroporated with shCTL, shMAD1 with RFP-tagged control vector, or shMAD1 with RFP-tagged c-MAD1 at E14.5 and analyzed at E17.5. Representative images (**c**) of migrating neurons. Schematic diagram of the length and orientation of leading processes (**d**, left) and statistical analysis (**d**, right) of the length of the leading process (shCTL, *n* = 48 cells; shMAD1, *n* = 34 cells; shMAD1 + c-MAD1, *n* = 32 cells). Representative images showing the orientation of cells transfected with shMAD1 (**e**, left) and quantification (**e**, right) of directionality (*n* = 3 brains for each group). Scale bars represent 100 μm (**a**) and 50 μm (**c**), respectively. Upper cortical plate (UCP); lower cortical plate (LCP); intermediate zone (IZ); sub-ventricular zone (SVZ); ventricular zone (VZ). Statistical analysis was conducted by using one-way ANOVA followed by Turkey’s post-hoc test. Analysis with nested model was conducted (**d**; Supplementary Dataset). Data are presented as means ± SEM. Statistical significance; *p < 0.05, **p < 0.01, ***p < 0.001, ****p < 0.0001 or n.s. (not significant).

Because E16 is the stage in which early newborn neurons actively migrate from the subventricular zone (SVZ) or ventricular zone (VZ) to the cortical plate (CP) during cortical development [37], we investigated the potential role of MAD1 in neuronal migration using in utero electroporation (IUE). MAD1 shRNA was injected into the developing cortex at E14.5 in utero, and brains were harvested at E18.5 (Fig. 1a, Supplementary Fig. 1c–g). We analyzed the compositions of transfected neurons migrating from SVZ/VZ to CP by 3D z-stacking. Notably, a significant migration defect was evidenced by the accumulation of migrating newborn neurons in the lower cortical plate (LCP) of MAD1-knockdown brains (Fig. 1b).

Although the nuclear function of MAD1 in dividing cells is relatively well-characterized [22, 38], its cytoplasmic functions in post-mitotic neurons have not been elucidated. Thus, we aimed to determine whether the migration defects observed in MAD1-knockdown neurons are associated with the regulatory mechanism occurring in the cytoplasm. To this end, we constructed a cytoplasmic version of MAD1 (c-MAD1, 181-717 amino acids, [22]) lacking the N-terminus harboring the nuclear localization signal (NLS) and shRNA#1-targeting region (Supplementary Fig. 1h). We confirmed that it was localized only in the cytoplasm of HEK293 cells (Supplementary Fig. 1i). Next, we tested whether the knockdown effect of MAD1 in the developing brain could be reversed by c-MAD1 expression. Indeed, c-MAD1 co-expression with MAD1 shRNA was sufficient to attenuate the migration delay (Fig. 1a, b). These results indicate that MAD1 regulates neuronal migration in the developing brain via a cytoplasmic mechanism.

Migrating neurons exhibit saltatory movement with repetitive extension and retraction of the leading edge [23]. To further assess the cause of migration defects, we characterized the morphology of migrating neurons in detail. After IUE at E14.5, brains were harvested at E17.5 to observe migrating neurons (Fig. 1c). After acquiring the brain slices, we used confocal microscopy for slice imaging and compared each z-stack image with the whole 3D-projection image to discriminate fluorescence-labeled neurons for further analysis. We measured the length of the leading process and its angle relative to the direction of migration toward the pia mater (Fig. 1d, left). MAD1-knockdown neurons exhibited a shorter leading process than control neurons (Fig. 1d, right) and a deviated angle of the leading process relative to the direction of migration (Fig. 1e), which likely accounted for the neuronal migration defects upon MAD1 knockdown. The neuronal migration defects were efficiently rescued by co-expression of an shRNA-resistant form of full-length MAD1 (Supplementary Fig. 2a–d).

We further characterized whether these migration defects are related to the proliferation of neural progenitor cells or neuronal cell fate determination [14, 18–21]. We conducted IUE at E13.5 followed by BrdU (Bromodeoxyuridine) injection at E14.5 and analyzed the alteration of BrdU-positive, proliferative cells at E15.5 [26, 39, 40]. As a result, BrdU-positive cells were significantly decreased upon MAD1-knockdown, indicating the roles of MAD1 in cell division as previously demonstrated. However, unlike full-length MAD1, c-MAD1 did not rescue the altered BrdU-positive cells caused by MAD1 knockdown (Supplementary Fig. 3a, b), in contrast to migration phenotypes (Fig. 1b, d, e). These phenomena suggest that the cell proliferation step requires intact MAD1 function but the neuronal migration mainly depends on cytoplasmic MAD1. We also examined whether the migration defects could be extended to the changes in neuronal cell fate determination. We immunostained brain sections for SOX2, a neural progenitor marker, and NeuN, a neuronal marker, to detect the changes in cell composition [26, 41, 42]. The fractions of SOX2-positive or NeuN-positive cells were not changed significantly (Supplementary Fig. 3c–f), indicating that MAD1 is less likely to be directly involved in cell fate determination.

### MAD1-deficiency causes abnormal Golgi–centrosome positioning in migrating neurons

**Fig. 2:**
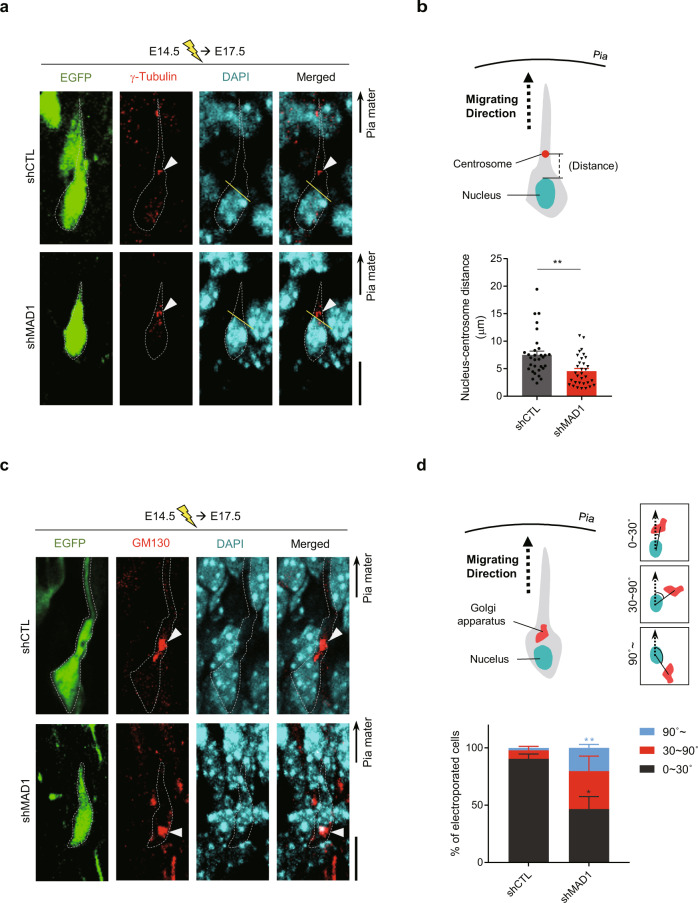
Deficiency of MAD1 causes alteration of Golgi-centrosome positioning. **a**, **b** Mouse embryos were electroporated with shCTL or shMAD1 at E14.5 and analyzed at E17.5. Representative images of EGFP-positive shCTL- or shMAD1-transfected neurons co-immunostained with γ-tubulin (red). EGFP-only images (leftmost) were acquired via z-stacking and z-projection to visualize the cell surface. Arrowheads point to the centrosome, and straight yellow lines demarcate the upper region of the nucleus. The cell surface is delineated with a white dashed line (**a**). Schematic diagram of the distance between the nucleus and centrosome (**b**, upper) and statistical analysis (**b**, lower) of length (shCTL, *n* = 31 cells; shMAD1, *n* = 32 cells). **c**, **d** Representative images of EGFP-positive shCTL- or shMAD1-transfected neurons co-immunostained with GM130 (red). Arrowheads point to Golgi, and dashed lines delineate the cell surfaces (**c**). Schematic diagram of assessing the position of Golgi with examples of categorization by the angle between migrating direction (dot arrow) and nucleus-Golgi alignment (straight line, **d**, upper). Statistical analysis of the fraction of cells classified with angle groups (**d**, lower, shCTL, *n* = 3 brains; shMAD1, *n* = 3 brains). Statistical significance (*) was assessed by comparison between shCTL and shMAD1 within the same angle group. Scale bars represent 10 μm (**a** and **c**). Statistical analysis was conducted by using Student’s *t*-test. Analysis with nested model was conducted (**b**; Supplementary Dataset). Data are presented as means ± SEM. Statistical significance; *p < 0.05, **p < 0.01, ***p < 0.001 or ****p < 0.0001.

Next, we evaluated the intracellular consequences of MAD1-deficiency in migrating neurons. As proper positioning of the centrosome is critical for the migration processes of neurons, we analyzed the distance between the nucleus and centrosome, which is a hallmark of migration fidelity during nucleokinesis [26, 27, 32, 43]. For this, we conducted IUE at E14.5, and embryonic brains were harvested at E17.5. Brain sections were immunostained with γ-tubulin centrosome marker and GM130 *cis*-Golgi marker. Since brain tissues are densely compacted with numerous cells, it is relatively tricky to analyze directly in the 3D projection image. Therefore, we followed the EGFP signal, a morphology marker, to discriminate electroporated cells. Immunostained individual cells were traced and analyzed by comparing each z-stack confocal image and 3D projection images. Immunohistochemical analyses revealed that MAD1 knockdown decreased the nucleus–centrosome distance in migrating neurons (Fig. 2a, b). Interestingly, when stained with GM130, the positioning of Golgi apparatuses located in the proximity of the centrosome was significantly altered by MAD1 knockdown (Fig. 2c). For quantification, we measured the position of the Golgi apparatus relative to the direction of migration (Fig. 2d, upper). While most neurons in the control group exhibited a Golgi position within a 0–30° range from the direction of migration (Fig. 2d, lower), the MAD1 knockdown group exhibited a deviation of Golgi from the appropriate position for normal migration. Indeed, centrosome-Golgi apparatus positioning determines cellular polarity critical for neuronal migration [23, 44–47], and abnormal morphologies or positioning of Golgi were reported in defects of neuronal migration [26, 48, 49].

Migrating neurons show dilation of the cytoplasmic region in conjunction with the movement of the nucleus [50, 51]. Therefore, we analyzed the potential cytoplasmic dilation of migrating neurons in MAD1-knockdown IUE brains. We measured the distance (*D*) from the upper tip of the nucleus and compared it with the nucleus radius (*r*, half of the major axis, Supplementary Fig. 4a). In this analysis, MAD1-knockdown groups show comparable value with control, indicating that the MAD1 knockdown effect is relatively specific to the length and directionality of the leading processes (Supplementary Fig. 4b, c). We also attempted to trace the multipolar-to-bipolar transition in the E16.5 neocortex IUEed at E14.5 [47, 52, 53]. The multipolar cells in the lower sub-ventricular zone and bipolar cells above the sub-ventricular zone at E16.5 were analyzed. In this analysis, we observed that the multipolar-to-bipolar transition is also affected by the MAD1 knockdown (Supplementary Fig. 4d, e). This result is consistent with our observation (Fig. 1c–e and Fig. 2) because this transition is also likely to require the proper regulation of dynamic Golgi/centrosomal positioning modulated by MAD1, further supporting the idea that MAD1 is a critical factor in establishing neuronal polarity contributed by Golgi-centrosomal positioning. [47, 52–54].

### MAD1 is critical for neurite outgrowth

**Fig. 3:**
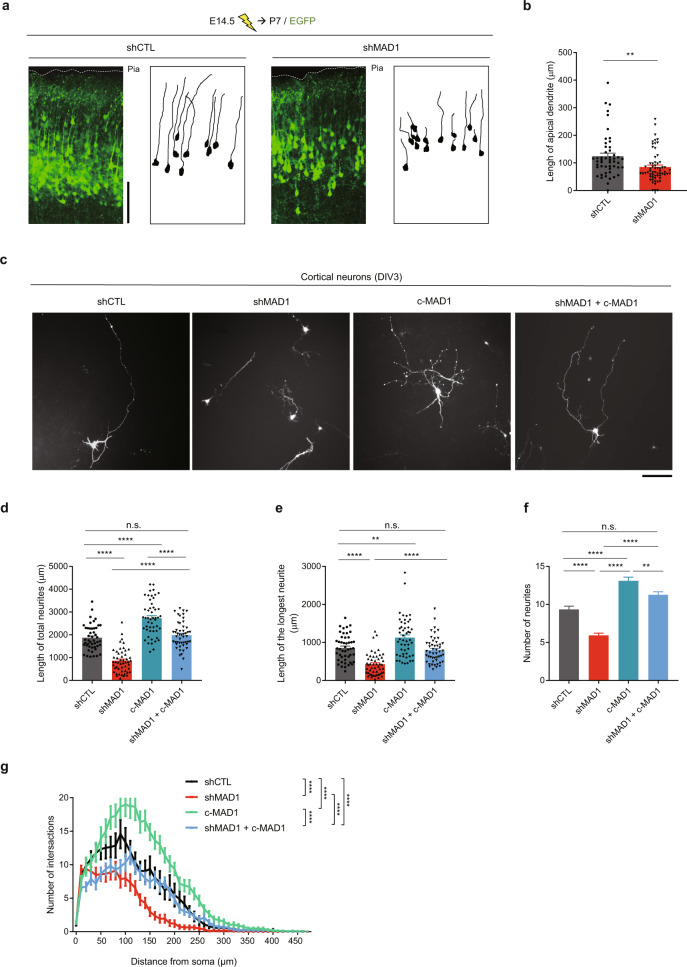
MAD1 regulates neurite outgrowth. **a**, **b** Mouse embryos were electroporated with shCTL or shMAD1 at E14.5 and analyzed at P7. Images were acquired by z-stacking and 3D projection by confocal microscopy. Only EGFP-positive neurons were analyzed, excluding neurons having fragmented structures inevitably damaged during the tissue sectioning. Representative images from each group with traced diagrams (**a**) and statistical analysis (**b**, shCTL, *n* = 50 cells; shMAD1, *n* = 62 cells) of the apical dendrite length are shown. Dashed lines demarcate pia mater (**a**). **c**–**g** Primary cultured mouse neurons were transfected at 1 day in vitro (DIV1) and analyzed at DIV3. Representative images from each group (**c**) and statistical analysis of total neurite length (**d**), longest neurite length (**e**), and the number of neurites (**f**, shCTL, *n* = 47 cells; shMAD1, *n* = 50 cells; c-MAD1, *n* = 50 cells; shMAD1 + c-MAD1, *n* = 50 cells). Sholl analysis of neurite outgrowth (**g**, shCTL, *n* = 30 cells; shMAD1, *n* = 40 cells; c-MAD1, *n* = 32 cells; shMAD1 + c-MAD1, *n* = 34 cells). Scale bars represent 100 μm (**a**) and 50 μm (**c**), respectively. Student’s *t*-test was used for statistical significance (**b**). One-way ANOVA (**d**–**f**) and two-way ANOVA (**g**) followed by Turkey’s post-hoc test were used. Analysis with nested model was conducted (**b** and **d**–**f**; Supplementary Dataset). Data are presented as means ± SEM. Statistical significance; *p < 0.05, **p < 0.01, ***p < 0.001, ****p < 0.0001 or n.s. (not significant).

After neuronal migration, the leading process becomes an apical primary dendrite, and the trailing process develops into an axon [55–57]. Thus, we examined whether the polarity defects caused by MAD1 knockdown could be further extended to neuronal differentiation processes by analyzing the neuronal morphology at P7 after IUE of MAD1 shRNA at E14.5. After z-stacking and 3D z-projection, we evaluated intact EGFP-labeled neurons, which show clear neurites and soma in the same slice. The length of P7 primary apical dendrites was reduced in the MAD1-knockdown group, suggesting that neurite outgrowth is also affected by MAD1-deficiency (Fig. 3a, b). Defects of neurite outgrowth were also observed in P14 brains (Supplementary Fig. 5a–c), suggesting that defects of neuronal polarity can result in abnormality of neuronal differentiation in later stages. To verify the cell autonomy of the phenotype, we cultured neurons from embryonic brains after IUE of MAD1 shRNA and observed a consistent reduction of the initial neurite length of the transfected neurons (Supplementary Fig. 5d, e). Next, we examined the effect of MAD1 knockdown in cultured primary neurons in more detail. Cultured cortical neurons were transfected with MAD1 shRNA at day 1 in vitro (DIV1), and the neurite length and complexity were measured at DIV3 (Fig. 3c). Consistently, MAD1 knockdown reduced neurite outgrowth in terms of total length (Fig. 3d), length of the longest neurite (Fig. 3e), and the number of neurites (Fig. 3f). In addition, Sholl analysis revealed that MAD1 knockdown altered the complexity of neuronal processes (Fig. 3g). These morphological phenotypes were effectively reversed by co-overexpression of c-MAD1 (Fig. 3c–g) or shRNA-resistant full-length MAD1 (Supplementary Fig. 6a–d). Notably, c-MAD1 overexpression upregulated neurite outgrowth.

Neuronal polarization can also affect the axon specification at the initiation of neurite differentiation [53, 58, 59]. To further verify whether MAD1-knockdown can affect the axonal determination, we electroporated MAD1 shRNA in utero and applied the electroporated brains to the primary culture. 24 hr after plating, the initial neurite differentiation was analyzed by immunostaining with Tau, an axonal marker. In this analysis, we observed ‘no-axon’ neurons more frequently in the MAD1-knockdown group (Supplementary Fig. 7a, b). We also analyzed axonal specification of human neurons, differentiated 12 days from human neural progenitor cells. Consistently, the axonal determination was not affected in MAD1-knockdown groups (Supplementary Fig. 7c, d). ‘Multi-axon’ neurons were not observed in these experiments. Thus, abnormal polarity caused by MAD1-knockdown is more likely to delay differentiation of the neurite rather than change the fate of neurites.

### MAD1 regulates Golgi morphology and post-Golgi trafficking

**Fig. 4:**
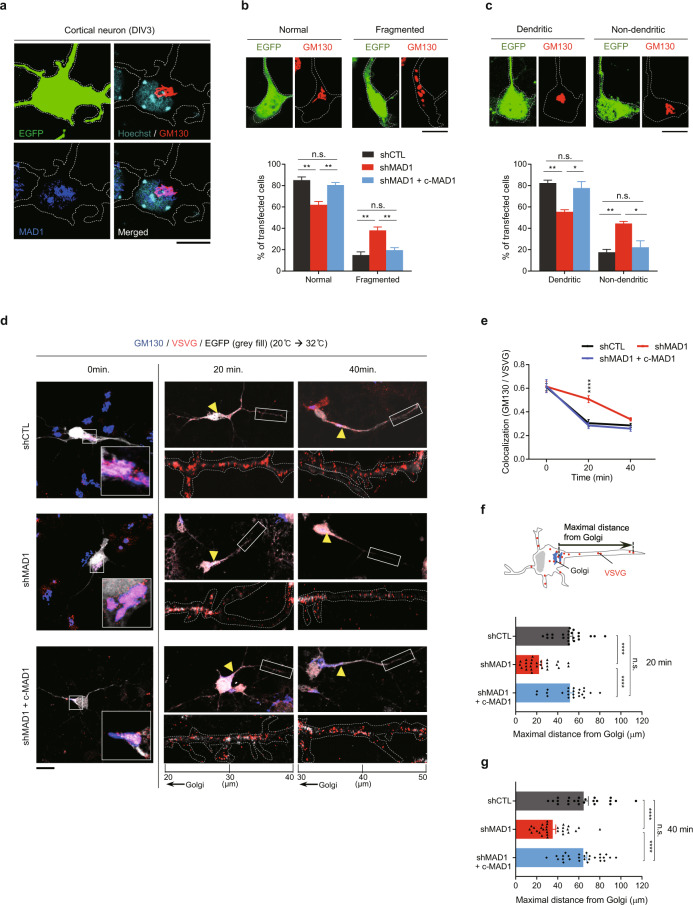
MAD1-deficiency changes Golgi morphology and post-Golgi trafficking. **a** Colocalization of endogenous MAD1 (blue) with GM130 (red) in neurons at DIV3. A saturated EGFP signal (green) was used as a morphological marker. Dashed lines demarcate the cell periphery. **b**, **c** The morphology of Golgi was assessed using z-stacking and z-projection of images immunostained with GM130 (red). EGFP (green) with a dashed line demarcates cell morphology. Representative images from the “Normal” and “Fragmented” groups (**b**, upper). Graph showing the percentage of transfected cells with each pattern of Golgi morphology (**b**, lower). Representative images of “Dendritic” or “Non-dendritic” Golgi position in DIV3 neurons (**c**, upper) and the percentage of transfected cells with each pattern of Golgi positioning (**c**, lower, shCTL, *N* = 3; shMAD1, *N* = 3; *n* > 30 cells for each *N*). **d**–**g** Neurons were transfected with VSVG + shCTL, VSVG + shMAD1, or VSVG + shMAD1 + c-MAD1. Neurons were co-immunostained with GM130 (blue) and VSVG (red) in a time-dependent manner (0, 20, and 40 min). EGFP signal shows the morphology of transfected cells (gray fill). Representative images of the soma region showing Golgi-accumulated VSVG at 0 min (**d**, left). Representative time-dependent images from each group showing whole neuronal morphology with enlarged neurites in the 20 μm range (20–40 μm from Golgi at 20 min, 30–50 μm from Golgi at 40 min). Arrowheads point to the Golgi (**d**). Statistical analysis of colocalization between GM130 and VSVG particles. Statistical significance (*) was assessed by comparing shCTL with shMAD1 at the same time point (**e**). A schematic diagram for the measurement of maximal VSVG moving distance from the Golgi (**f**, upper). Statistical analysis of maximal moving distance from Golgi at 20 min (**f**, lower) and 40 min (**g**, shCTL, 0 min, *n* = 22; 20 min, *n* = 23; 40 min, *n* = 23; shMAD1, 0 min, *n* = 17; 20 min, *n* = 27; 40 min, *n* = 29; shMAD1 + c-MAD1, 0 min, *n* = 14; 20 min, *n* = 23; 40 min, *n* = 27). Scale bars represent 10 μm (**a**–**d**). Statistical analysis was conducted by using one-way ANOVA followed by Turkey’s post-hoc test. Analysis with nested model was conducted (**e**–**g**; Supplementary Dataset). Data are presented as means ± SEM. Statistical significance; *p < 0.05, **p < 0.01, ***p < 0.001, ****p < 0.0001 or n.s. (not significant).

Previous studies demonstrated that MAD1 is localized to the nucleus, cytoplasm, and Golgi apparatus in non-neuronal cells [14, 18, 21, 22]. To investigate the roles of MAD1 in neurons, we performed immunocytochemistry for the endogenous MAD1 protein. We observed that MAD1 and c-MAD1 proteins were co-stained with GM130 (Fig. 4a and Supplementary Fig. 8a), which is consistent with a previous report showing the Golgi-localized fraction of MAD1 in the cell line [22]. The neuronal Golgi apparatus is positioned in a densely stacked pattern near the initiation site of the primary apical dendrite, with some scattered satellite Golgi within the apical dendrite (Supplementary Fig. 8b) [28]. Because knockdown of MAD1 may alter the proper positioning of Golgi (Fig. 2c, d), we monitored the morphology of Golgi apparatus in primary neurons. Neurons were transfected with MAD1 shRNA at DIV1, and the extent of abnormal fragmentation and location was analyzed at DIV3. MAD1 knockdown significantly increased the fraction of neurons with fragmented Golgi morphology (Fig. 4b, Supplementary Fig. 8c, d) and non-dendritic Golgi positioning (Fig. 4c). When we co-expressed c-MAD1 upon MAD1 knockdown, the defects were reversed (Fig. 4b, c). These findings indicate that MAD1 is localized to the Golgi apparatus and regulates its proper positioning, which is critical for neuronal polarity.

Next, we investigated whether abnormal Golgi morphology and positioning are connected to functional defects in the Golgi [29, 60]. We further examined whether MAD1 knockdown could alter the general trafficking of the Golgi. We employed a temperature-sensitive mutant version of vesicular stomatitis virus G protein (VSVG) to assess intracellular membrane trafficking [30, 61]. We transfected HeLa cells with human MAD1 shRNA and monitored whether membrane trafficking was affected. While the movement from the endoplasmic reticulum (ER) to the Golgi apparatus did not change, we observed a significant decrease in VSVG movement from the Golgi apparatus to the plasma membrane (Supplementary Fig. 8e, f), implying a specific role for MAD1 to the trafficking from the Golgi apparatus to the plasma membrane. Next, we applied this experimental scheme to primary cultured neurons. However, the cytotoxicity occasionally caused by VSVG and temperature stress could lower the neuronal conditions. Also, VSVG movements are relatively slow in the primary neurons than in immortalized cell lines. Therefore, we adjusted the experimental conditions focusing on post-Golgi trafficking (See also; Supplementary Methods). In the MAD1 knockdown group, fewer VSVG puncta were transported from the Golgi at 20 min or 40 min after shifting to 32 °C (Fig. 4d, e). The maximal distance of VSVG puncta from the Golgi was also reduced in the MAD1-knockdown group, reflecting impaired trafficking efficiency (Fig. 4f, g). Defects in MAD1-knockdown neurons were restored by co-expression of c-MAD1 (Fig. 4d–g). These data demonstrate that MAD1 localizes to the Golgi apparatus and is necessary for normal Golgi morphology and trafficking function, which are required for establishing neuronal polarity.

### MAD1 functionally interacts with the KIFC3 motor

**Fig. 5:**
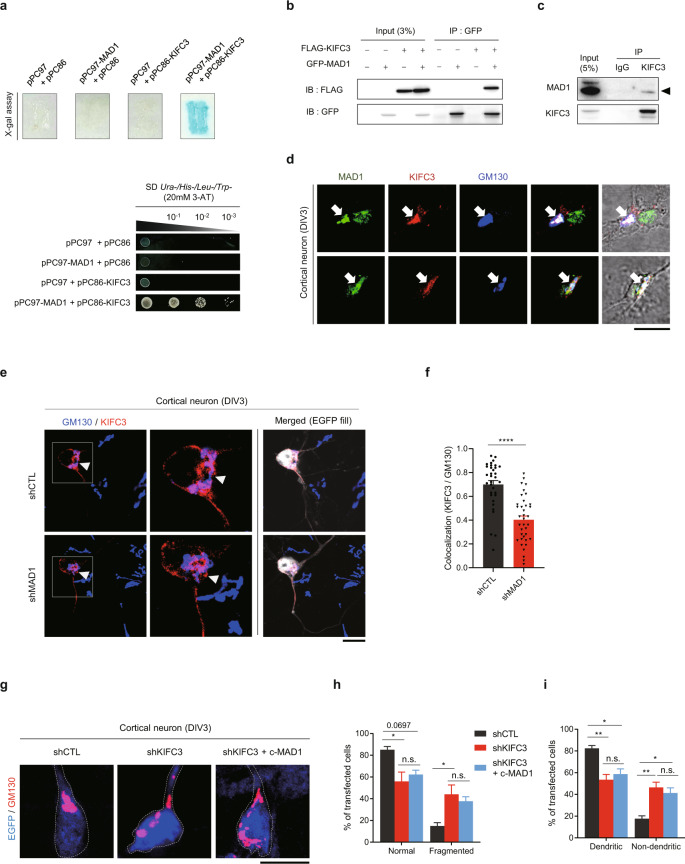
MAD1 physically and functionally interacts with KIFC3. **a** Interaction of MAD1 and KIFC3 shown by X-gal assay (upper) and growth on SD *Ura-/His-/Leu-/Trp*- media (lower). **b** Co-immunoprecipitation (co-IP) result of GFP-MAD1 and FLAG-KIFC3 in HEK293 cells. IB; immunoblot. **c** Co-IP of endogenous MAD1 and KIFC3 in mouse E16 embryonic brain. Arrowhead points to MAD1 (83 kDa). **d** Endogenous colocalization of MAD1 (green) and KIFC3 (red) to Golgi (blue) in DIV3 cortical neurons with DIC images showing cell morphologies. Arrows point to Golgi region. **e, f** Representative images showing localization of KIFC3-RFP to Golgi (**e**) and quantification of colocalization between KIFC3-RFP and GM130 (**f**, shCTL, *n* = 33; shMAD1, *n* = 37). Arrowheads point to Golgi region. **g**–**i** Cortical neurons were transfected with shCTL, shKIFC3, or shKIFC3 with c-MAD1. The morphology and positioning of Golgi were assessed by immunostained with GM130 (red). EGFP (blue) with dashed line demarcates cell morphology. Representative images from each group (**g**) and percentage of cells showing altered Golgi morphology (**h**) and positioning (**i**, shCTL, *N* = 3; shMAD1, *N* = 3; *n* > 30 cells for each *N*). Scale bars represent 10 μm (**d**, **e**, and **g**). Student’s *t*-test was used for statistical significance (**f**). One-way ANOVA followed by Turkey’s post-hoc test was used (**h** and **i**). Analysis with nested model was conducted (**f**; Supplementary Dataset). Data are presented as means ± SEM. Data are presented as means ± SEM. Statistical significance; *p < 0.05, **p < 0.01, ***p < 0.001, ****p < 0.0001 or n.s. (not significant).

To identify the mediator of MAD1 function in neurons, we conducted yeast-two-hybrid screening against a human fetal brain library using human *MAD1L1* as bait. We identified several positive interactors. Among the positives, we focused on the kinesin-like protein KIFC3 (kinesin family member C3) because it is a microtubule-minus-end-directed motor protein with a function for neuronal morphogenesis [62, 63] and plays a critical role in the assembly of Golgi stacks [64]. KIFC3 exhibited a strong interaction with MAD1 in the yeast-two-hybrid assay, as evidenced by the X-gal assay and growth on selective media (Fig. 5a). This interaction was also confirmed by co-immunoprecipitation of MAD1 and KIFC3 (Fig. 5b, c).

Next, we tested the potential collaboration of MAD1 and KIFC3 in regulating Golgi morphology and neuronal polarity. In the ICC analysis, KIFC3 and MAD1 are co-localized at the Golgi (Fig. 5d), suggesting that their physical interaction is linked to Golgi function. Interestingly, when MAD1 was depleted by shRNA, colocalization of KIFC3 and Golgi marked by GM130 was significantly reduced, suggesting a role for MAD1 in the recruitment of KIFC3 to the Golgi complex (Fig. 5e, f). Also, neurite outgrowth was assessed by introducing KIFC3 shRNA with c-MAD1. As a result, the knockdown of KIFC3 significantly affected the outgrowth of neurites by decreasing their length, number, and complexity (Supplementary Fig. 9). The c-MAD1 co-expression with KIFC3 shRNA failed to alter the KIFC3 knockdown effect, and the double-knockdown of MAD1 and KIFC3 did not further reduce neurite length compared with the single knockdown of MAD1 or KIFC3 (Supplementary Fig. 10a–d), supporting the collaboration of MAD1 and KIFC3.

We also analyzed the morphology and positioning of the Golgi apparatus. When KIFC3 shRNA was introduced into developing primary neurons, the fraction of cells with fragmented and/or abnormally positioned Golgi significantly increased (Fig. 5g–i), and this change was not attenuated by co-expression of c-MAD1. These findings indicate that MAD1 regulates the Golgi apparatus by recruiting KIFC3 to the Golgi.

### MAD1 and KIFC3 collaborate to regulate neuronal polarity

**Fig. 6:**
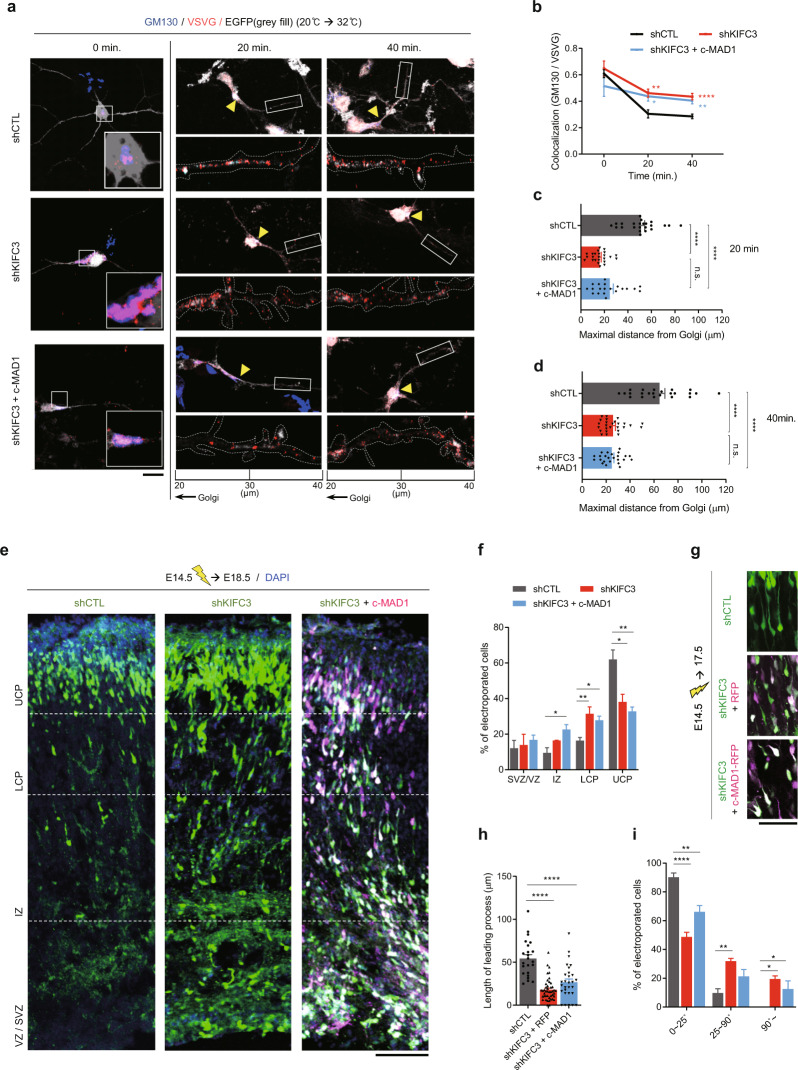
MAD1 and KIFC3 collaborate to regulate neuronal polarity. **a**–**d** Primary cultured neurons were transfected with VSVG + shCTL, VSVG + shKIFC3, or VSVG + shKIFC3 + c-MAD1. Neurons were fixed and co-immunostained with GM130 (blue) and VSVG (red) in a time-dependent manner (0, 20, and 40 min). Representative images of the soma region showing Golgi-accumulated VSVG at 0 min (**a**, left). Representative time-dependent images from each group showing whole neuronal morphology with enlarged neurites in the 20 μm range (20–40 μm from Golgi). Arrowheads indicate the Golgi region (**a**). Statistical analysis of colocalization between GM130 and VSVG particles. Statistical significance (*) was assessed by comparing shCTL with shKIFC3 or shKIFC3 + c-MAD1 at 20 min or 40 min (**b**). Statistical analysis of maximal moving distance from the Golgi at 20 min (**c**) and 40 min (**d**, shCTL, 0 min, *n* = 22; 20 min, *n* = 23; 40 min, *n* = 23; shKIFC3, 0 min, *n* = 14; 20 min, *n* = 24; 40 min, *n* = 26; shKIFC3 + c-MAD1, 0 min, *n* = 9; 20 min, *n* = 20; 40 min, *n* = 24). **e**, **f** Mouse embryos were electroporated with shCTL, shKIFC3, or shKIFC3 with RFP-tagged c-MAD1 at E14.5 and analyzed at E18.5. Transfected cells are GFP-positive for shCTL or shKIFC3 and RFP-positive (magenta) for c-MAD1. Representative images from each group (**e**) and statistical analysis of neuronal migration (**f**, shCTL, *n* = 4 brains; shKIFC3, *n* = 3 brains; shKIFC3 + c-MAD1, *n* = 3 brains). **g**–**i** Mouse embryos were electroporated with shCTL, shKIFC3, or shKIFC3 with RFP-tagged c-MAD1 at E14.5 and analyzed at E17.5. Representative images from each group (**g**), and statistical analysis of leading process length (**h**, shCTL, *n* = 23; shKIFC3, *n* = 44; shKIFC3 + c-MAD1, *n* = 29) and directionality (**i**, shCTL, *n* = 3 brains; shKIFC3, *n* = 4 brains; shKIFC3 + c-MAD1, *n* = 4 brains). Scale bars represent 10 μm (**a**), 100 μm (**e**) and 50 μm (**g**), respectively. Statistical analysis was conducted by using one-way ANOVA followed by Turkey’s post-hoc test. Analysis with nested model was conducted (**b**–**d** and **h**; Supplementary Dataset). Data are presented as means ± SEM. Statistical significance; *p < 0.05, **p < 0.01, ***p < 0.001, ****p < 0.0001 or n.s. (not significant).

Next, we verified whether post-Golgi trafficking is also regulated by MAD1-KIFC3. When vesicular trafficking was examined using temperature-sensitive VSVG, trafficking from the Golgi to the plasma membrane, as measured by the number of GM130-positive VSVG puncta (Fig. 6a, b) and maximal distance of VSVG puncta from the Golgi apparatus (Fig. 6c, d), was significantly downregulated upon KIFC3 knockdown as seen in MAD1-knockdown condition. Consistently, defects in Golgi morphology and trafficking upon the loss of KIFC3 were not reversed by co-overexpression of c-MAD1 (Fig. 6a–d), indicating that MAD1 regulates Golgi through KIFC3.

Finally, we examined whether MAD1 and KIFC3 are functionally related in the MAD1-relevant neuronal development process in vivo. We injected KIFC3 shRNA into the developing brain at E14.5 via IUE and analyzed the brain at E18.5. The KIFC3 knockdown group exhibited more significant cell accumulation in the LCP than the control group (Fig. 6e, f), a sign of delay in neuronal migration, as observed in the MAD1-knockdown condition. The length and direction of leading processes were also significantly affected (Fig. 6g–i). Knockdown of KIFC3 notably decreased the length of the leading process (Fig. 6h) and changed the polarity of migrating neurons (Fig. 6i). Consistent with findings observed in cultured neurons in vitro, these defects were not restored by c-MAD1 co-expression, indicating the dependency of MAD1 functions in these processes on KIFC3 (Fig. 6e–i). Additionally, the morphology defects of migrating neurons are not further exacerbated in double-knockdown of MAD1 and KIFC3 compared with individual knockdown conditions (Supplementary Fig. 10e–g), which is consistent with the polarity analysis.

### MAD1 regulates the development of human neurons and organoids

**Fig. 7:**
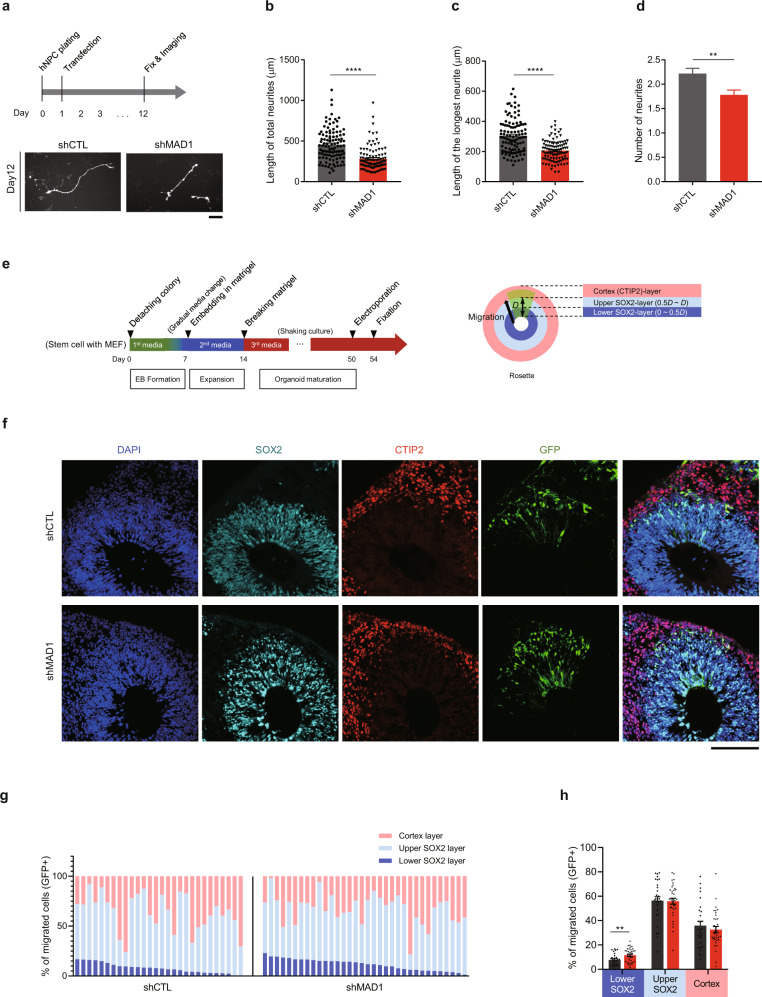
MAD1-deficiency impairs the development of neurons and organoids derived from human stem cells. **a**–**d** Initial neurite outgrowth analysis in human neurons. A schematic diagram of human neuron culture from hNPC (**a**, upper) and representative images of day 12 neurons transfected with shCTL or shMAD1 (**a**, lower) are shown. The length of total neurites (**b**), length of the longest neurite (**c**), and the total number of neurites (**d**) were analyzed (shCTL, *n* = 110; shMAD1, *n* = 101). **e**–**h** Neuronal migration analysis in human cerebral organoids. A schematic diagram of human cerebral organoid culture is shown (**e**, left). Rosette cell layers are demarcated by immunostaining for SOX2, a neural progenitor marker, and CTIP2, a cortical layer marker (**e**, right). Representative images of electroporated rosettes after immunostaining are shown (**f**). Locations of migrated cells in organoids located in the cortical layer (light red), upper SOX2 layer (light blue), and lower SOX2 layer (blue) are aligned by the decreasing number of cells in the lower SOX2 layer (**g**). The fraction of the migrated cells in the indicated layers was analyzed (**h**, shCTL, *n* = 28; shMAD1, *n* = 34). Scale bars represent 10 μm (**a**, lower) and 100 μm (**f**). Statistical analysis was conducted by using the unpaired two-tailed Student’s *t*-test. Analysis with nested model was conducted (**b**–**d**; Supplementary Dataset). Data are presented as means ± SEM. Statistical significance; *p < 0.05, **p < 0.01, ***p < 0.001, ****p < 0.0001 or n.s. (not significant).

Finally, we attempted to recapitulate the main findings in neurons and cerebral organoids derived from human stem cells [65]. We characterized the neurons differentiated from human neural progenitor cells (hNPC) for measuring the initial neurite on day 12 (Fig. 7a). Similar neurite outgrowth defects were observed by knockdown of MAD1 (Fig. 7b–d). Neuronal migration was also monitored in human cerebral organoids [66]. We electroporated organoids on day 50 with scrambled shRNA and human MAD1 shRNA and analyzed the neuronal migration on day 54 (Fig. 7e, left) using CTIP2 and SOX2 as cortical and neural progenitor markers, respectively (Fig. 7e, right) [41, 66–69]. In this condition, MAD1 knockdown mildly but significantly impaired neuronal migration (Fig. 7f). When the migration index was analyzed along with immunostaining of CTIP2 and SOX2 layer markers, more neurons were accumulated in lower SOX2-layers (Fig. 7g, h). These results recapitulate the observations in developing mouse neocortex and further support the notion that *MAD1L1* is a critical regulator of human neuronal development.

## Discussion

**Fig. 8:**
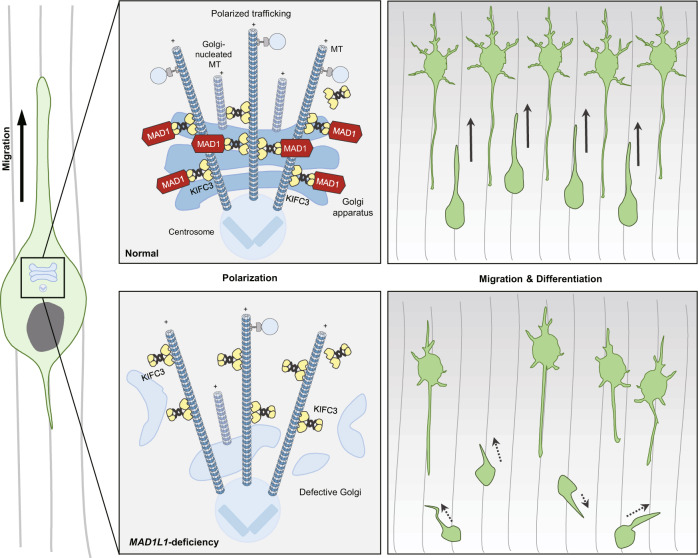
MAD1 and KIFC3 regulate neuronal development. Migrating neurons extend a leading process to the cortical surface and a trailing process opposite to the direction of migration. The trailing process subsequently develops into the axon. MAD1 recruits microtubule-minus-end-directed KIFC3 to the Golgi apparatus, thereby regulating Golgi assembly and functionality (upper). The deficiency of *MAD1L1* fails to recruit KIFC3 to the Golgi apparatus and disrupts neuronal polarity (lower). This polarity determination during migration is critical for neuronal morphogenesis and brain development (MT microtubule).

In this study, we demonstrated the role of the SZ-associated gene *MAD1L1* in neocortical development. *MAD1L1* has been repeatedly identified as one of the genes most strongly associated with SZ in multiple GWAS and methylation quantitative trait loci (meQTL) studies [7–12]. However, no studies have reported its direct involvement in the nervous system to date. Our data reveal a novel function of MAD1 in nucleokinesis during neurodevelopment. Nucleokinesis is composed of multiple steps that involve centrosome and Golgi positioning ahead of the moving nucleus toward the leading edge [47, 55]. During this process, the leading edge extends toward the direction of migration and further develops into dendrites. The trailing process, which is the protrusion of the opposite side of the migrating neuron, develops into the initial axon [57]. These phenomena indicate that migration and axonal differentiation coincide during development in conjunction with polarity determination. According to our model, MAD1 contributes to the formation of leading processes and subsequent neuronal migration and morphogenesis by regulating the integrity of the Golgi complex (Fig. 8). Because proper migration and differentiation are the initial processes of arraying neurons in appropriate regions, they are also critical for subsequent processes, including synaptogenesis, dendritic arborization, and neuronal type determination required for functional neural circuits [70–73]. Previous reports also suggested that these events need to be temporally accurate to ensure proper brain development [2, 5, 37, 74, 75]. This notion may explain the significant defects of the axon or dendrite differentiation in MAD1-knockdown neurons. In this regard, the strong genetic association of *MAD1L1* with SZ supports the neurodevelopmental hypothesis of SZ, which argues that SZ occurs due to altered brain structure, abnormal neuronal composition in the forebrain regions, and/or layering defects in the neocortex [2, 74, 76].

Our data suggest that MAD1 is a crucial regulatory protein for maintaining the morphology and trafficking function of the Golgi complex. The Golgi apparatus is a vital organelle for protein sorting, modification, and transport. Neuronal migration and differentiation require dynamic changes in cell morphology. Directional transport of receptor proteins and membrane materials to the protrusion sites is necessary for dynamic cell shape remodeling [25, 45, 77, 78]. For example, the trafficking of VLDLR (Very low-density lipoprotein receptor) and LRP8 (Low-density lipoprotein receptor-related protein 8) involved in the reelin pathway is crucial for neuronal migration and morphogenesis [77, 79, 80]. Also, defective transport of AMPA (α-amino-3-hydroxyl-5-methyl-4-isoxazole-propionate) receptor or NMDA (N-methyl-D-aspartate) receptor to the postsynaptic sites can be linked to SZ pathology [2, 81–85]. These observations support the idea that post-Golgi trafficking can be associated with neuronal development and SZ vulnerability. Indeed, analyses of various SZ susceptibility factors support the links between the Golgi apparatus and SZ etiology. For instance, DISC1 (Disrupted-in schizophrenia 1), involved in multiple neurodevelopmental processes, localizes to the Golgi apparatus in neurons and astrocytes, supposedly regulating neurite outgrowth and synaptic vesicle transport [27, 31, 86]. Dysbindin (Dystrobrevin-binding protein 1), one of the BLOC-1 (Biogenesis of lysosome-related organelles complex 1) subunits, also interacts with the Golgi complex, and dysbindin-deficient mice exhibit reduced Golgi function in the hippocampus [87]. Together with our data, these findings support the importance of Golgi functionality in neuronal development and potentially in the etiology of SZ.

From a mechanistic standpoint, it is noteworthy that MAD1 and KIFC3 collaborate to regulate Golgi structure and function. In our model (Fig. 8), if KIFC3 is not recruited by MAD1 correctly, the Golgi complex is not assembled as a stable structure, as evidenced by its scattered morphology and impaired trafficking. These results provide plausible explanations for how MAD1 regulates neuronal development via the Golgi complex. First, MAD1 may affect the tethering of microtubules to the Golgi by interacting with KIFC3. Golgi stacks are assembled at the pericentrosomal regions, achieved by microtubule minus-end-directed motor proteins such as dynein [88] and KIFC3 [64]. Also, Golgi can act as a microtubule-organizing center (MTOC) and Golgi-nucleated microtubules grow toward the cell periphery [45, 89]. Golgi integrity is maintained by its tight association with microtubules, which is crucial for the establishment of polarized secretory pathways [90]. Upon MAD1-deficiency, we observed that KIFC3 localized to the microtubule minus-end and less with the Golgi apparatus indicating a critical role of MAD1 in the recruitment of KIFC3. Therefore, the physical interaction between MAD1 and KIFC3 may ensure the structural and functional association of microtubules and the Golgi complex, thereby supporting the proper formation and positioning of Golgi essential for neuronal polarity. Second, this notion can be extended to the role of MAD1 in polarized trafficking in developing neurons. We observed that loss of MAD1 and/or KIFC3 damaged Golgi morphology and impaired post-Golgi trafficking. In addition to the possibility that defective Golgi structures are indirectly responsible for the defects in post-Golgi trafficking by reducing cargo-motor association and transport efficiency, various motor proteins responsible for post-Golgi trafficking may also be affected by Golgi integrity. Indeed, previous reports have suggested that MAD1 interacts with microtubule-binding proteins such as kinesin motor CENP-E [18] and kinesin-like protein KIF9 (IntAct Molecular Interaction Database, https://www.ebi.ac.uk/legacy-intact). Members of the kinesin superfamily proteins differ in their functionalities and structures but share conserved domains [91]. Thus, MAD1 may participate in anterograde cargo transport as a general regulator of microtubule-binding motor proteins, which is required for the maintenance of the dynamic nature of neuronal polarity in the developing neocortex.

Finally, multiple GWAS revealed genetic associations of *MAD1L1* with bipolar disorder [9, 92] and major depressive disorder [93, 94], which encourage further investigations regarding the role of MAD1 in major psychiatric diseases. Our data shows that *MAD1L1*-deficiency can cause significant defects in human neurons and cerebral organoids, implying that our findings are relevant to the neuronal development in the human brain. Considering that brain development is an array of multiple processes, any defect at an early stage, such as neuronal migration, may result in deficiencies in the following steps, such as changes in neuronal connectivity or ultimate brain functionalities [70, 71, 95, 96]. Thus, understanding the functions of MAD1 during brain development will provide new insights into the etiology of major psychiatric and neurodevelopmental disorders.

## Supplementary information

Supplementary Figures and Legends

Supplementary Methods

Supplementary Dataset - nested statistics
