# Supplementary material for: Schizophrenia-associated Mitotic Arrest Deficient-1 (MAD1) regulates the polarity of migrating neurons in the developing neocortex
Source: Mol Psychiatry. 2022 Nov 10;28(2):856–70. doi: 10.1038/s41380-022-01856-5 (PMC9908555; doi:10.1038/s41380-022-01856-5)
Supplement: Supplementary file 1 — Supplementary Figures and Legends [file 41380_2022_1856_MOESM1_ESM.pdf]

Supplementary Information

Schizophrenia-associated Mitotic Arrest Deficient-1 (MAD1) regulates the polarity of migrating neurons in the developing neocortex

Bon Seong Goo, Dong Jin Mun, Seunghyun Kim, Truong Thi My Nhung, Su Been Lee, Youngsik Woo, Soo Jeong Kim, Bo Kyoung Suh, Sung Jin Park, Hee-Eun Lee, Kunyou Park, Hyunsoo Jang, Jong-Cheol Rah, Ki-Jun Yoon, Seung Tae Baek, Seung-Yeol Park*, and Sang Ki Park*

***Correspondence:** seungpark@postech.ac.kr, skpark@postech.ac.kr

Supplementary Figure 1.

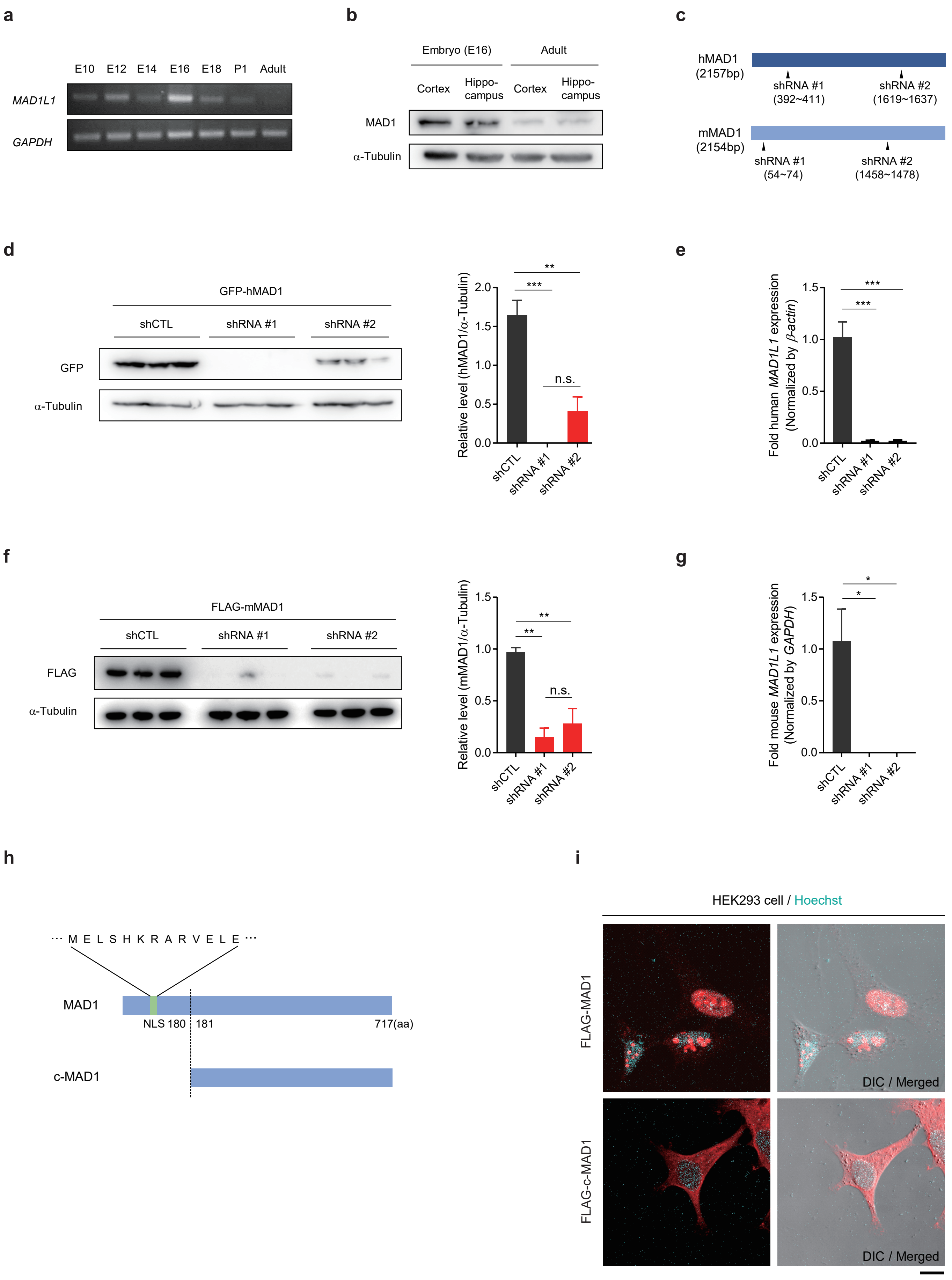

Supplementary Figure 1. Expression profile of *MAD1L1* in the developing brain and generation of shRNA constructs

(a) Semi-quantitative RT-PCR analysis showing endogenous *MAD1L1* expression in the mouse brain. *GAPDH* was used as a control. (b) Western blot result showing endogenous MAD1 protein in embryonic day 16 (E16) and adult mice. α-Tubulin was used as a control. (c) Schematic diagram of shRNA target regions for human MAD1 (hMAD1) [1, 2], and mouse MAD1 (mMAD1). (d) Western blot analysis showing the efficiencies of hMAD1 shRNAs (left) and quantification (right). Scrambled shRNA (shCTL) was used as a negative control. (e) Real-time PCR data showing the efficiencies of hMAD1 shRNAs (n = 3 for each group). (f) Western blot result showing the efficiencies of mMAD1 shRNAs (left) and quantification (right). (g) Real-time PCR result showing the efficiencies of mMAD1 shRNAs (n = 3 for each group). (h) Schematic diagram of mouse full-length MAD1 and c-MAD1. (i) Mouse FLAG-MAD1 and FLAG-c-MAD1 expression pattern in HEK293 cells. DIC images show the morphology of cells. Scale bar represents 10 μm (i). HEK293 cells (d-f, and i) and Neuro-2a cells (g) were used. Statistical significance was assessed by one-way ANOVA followed by Turkey’s post-hoc test (d-g). Data are presented as means ± SEM. Statistical significance: *p<0.05, **p<0.01, ***p<0.001, ****p<0.0001 or n.s. (not significant).

Supplementary Figure 2.

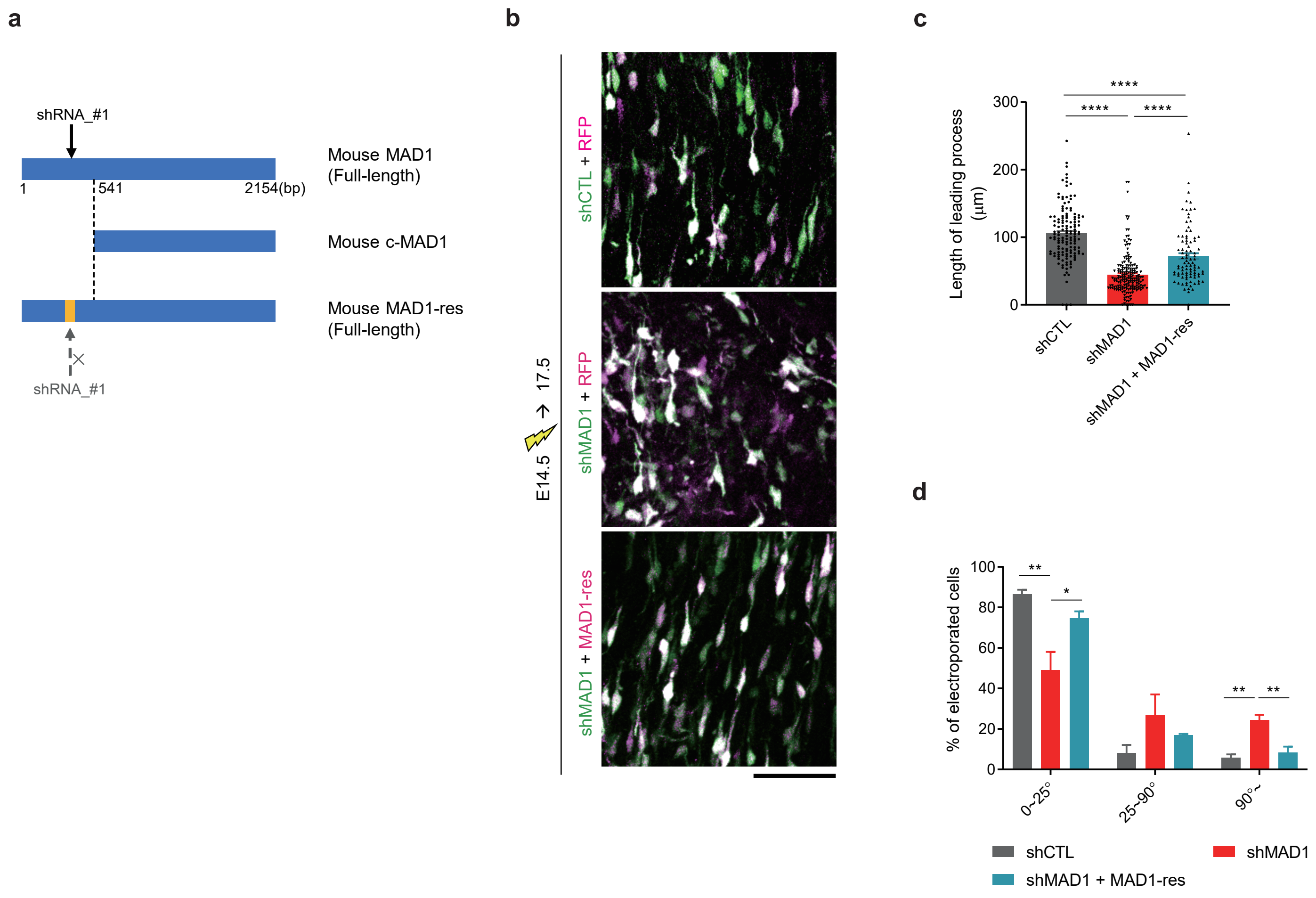

Supplementary Figure 2. Rescue of MAD1-deficiency with an shRNA-resistant form of MAD1

(a) Schematic diagram comparing full-length mouse MAD1, c-MAD1, and MAD1-res (shRNA #1 – resistant form of MAD1) cDNAs. shRNA #1 target regions are indicated. (b-d) Mouse embryos were electroporated at E14.5 with constructs as indicated and brains were analyzed at E17.5. Representative images (b). Length of leading process (c, shCTL, n = 140; shMAD1, n = 178; shMAD1+MAD1-res, n = 93) and directionality (d, shCTL, n = 3; shMAD1, n = 3, shMAD1+MAD1-res, n = 3). Scale bar represents 50 µm (b). Statistical significance was assessed by one-way ANOVA followed by Turkey's post-hoc test (c and d). Analysis with nested model was conducted (c; Supplementary Dataset). Data are presented as means ± SEM. Statistical significance: **p*<0.05, ***p*<0.01, ****p*<0.001 or *****p*<0.0001.

Supplementary Figure 3.

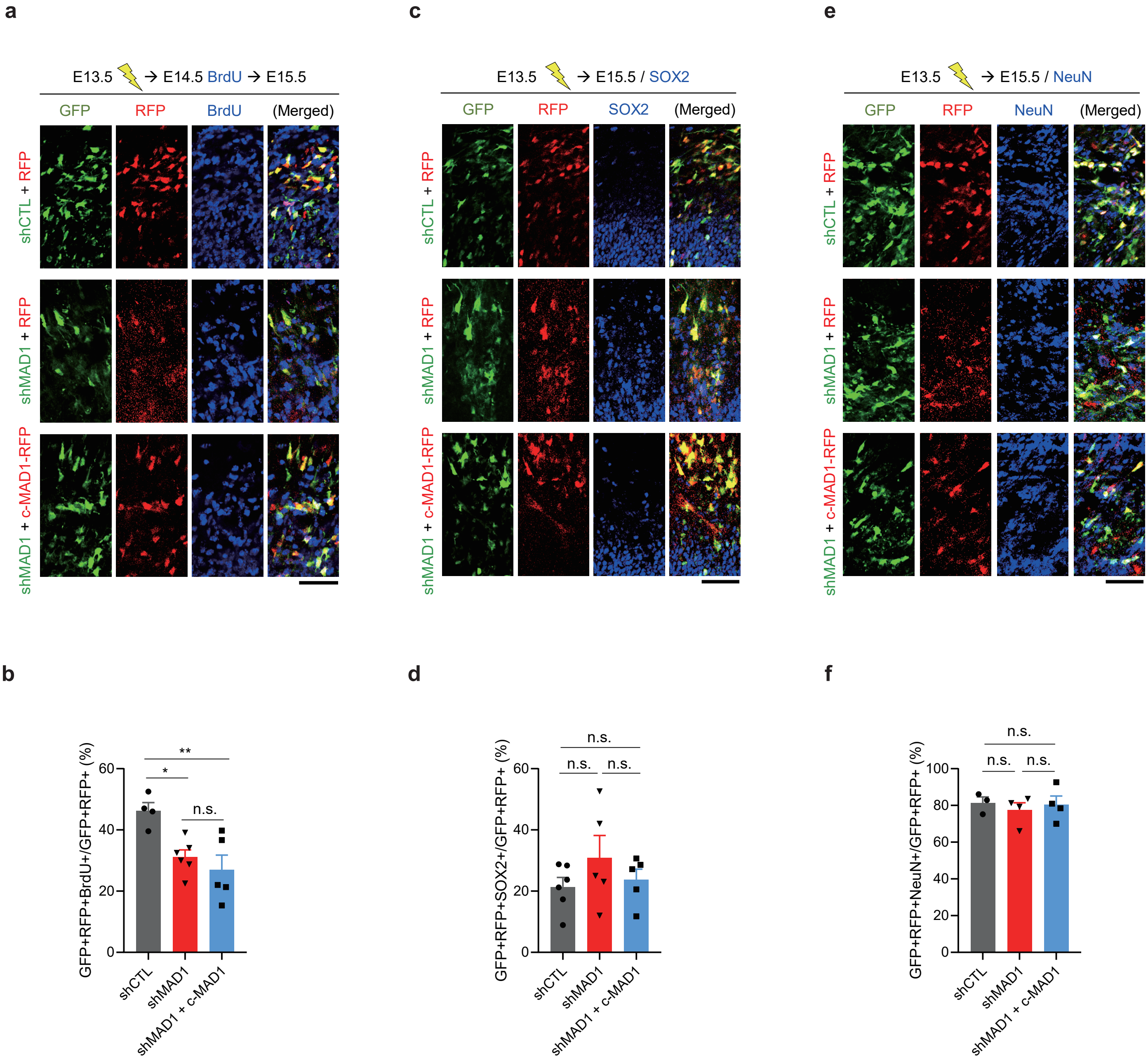

Supplementary Figure 3. Effects of MAD1-deficiency on NPC proliferation and neuronal differentiation.

(a-b) Mouse embryos were electroporated at E13.5 with constructs as indicated. BrdU (blue) was injected at E14.5 to mark cell proliferation. Representative images of each group (a). GFP/RFP/BrdU-positive cells were analyzed in E15.5 brains (b, shCTL, n = 4; shMAD1, n = 6; shMAD1+c-MAD1, n = 5). (c-d) Mouse embryos were electroporated at E13.5 with constructs as indicated. SOX2 (blue) was used to mark neural progenitor cells. Representative images of each group (c). GFP/RFP/SOX2-positive cells were analyzed in E15.5 brains (d, shCTL, n = 6; shMAD1, n = 5; shMAD1+c-MAD1, n = 5). (e-f) Mouse embryos were electroporated at E13.5 with constructs as indicated. NeuN (blue) was used to mark neurons. Representative images of each group (e). GFP/RFP/NeuN-positive cells were analyzed in E15.5 brains (f, shCTL, n = 3; shMAD1, n = 4; shMAD1+c-MAD1, n = 4). Scale bars represent 50 μm (a, c, and e). Statistical significance was assessed by one-way ANOVA followed by Turkey's post-hoc test (b, d, and f). Data are presented as means ± SEM. Statistical significance: *p<0.05, **p<0.01, ***p<0.001, ****p<0.0001 or n.s. (not significant).

Supplementary Figure 4.

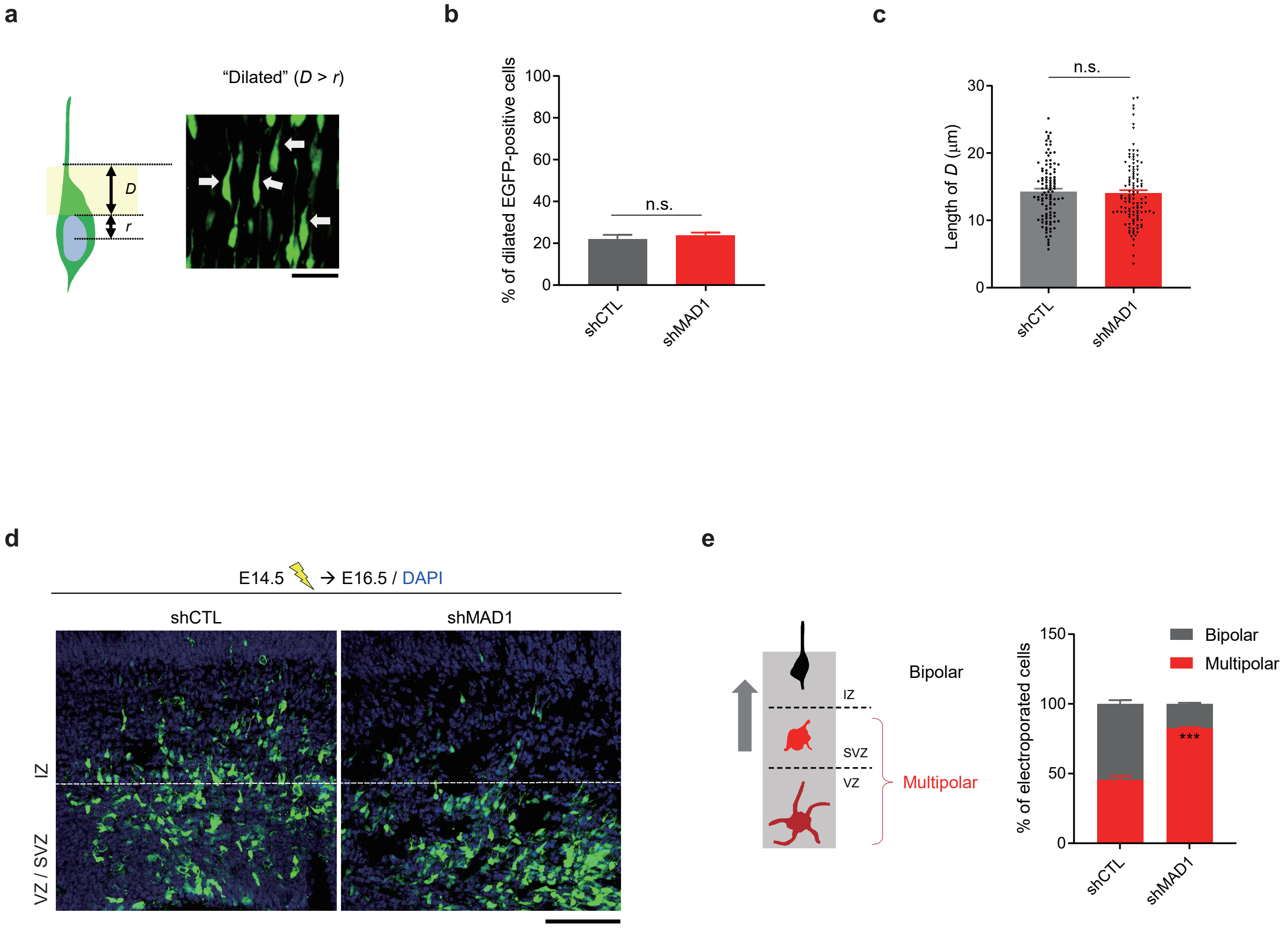

Supplementary Figure 4. Effects of MAD1-deficiency on cytoplasmic dilation and multipolar-to-bipolar transition

(a-c) Analysis of cytoplasmic dilation. Mouse embryos were electroporated at E14.5 with constructs as indicated and brains were analyzed at E17.5. Half of the major axis of the nucleus (*r*) and cytoplasmic distance from the upper tip of the nucleus (*D*) of migrating neurons were compared (a, left). Arrows in a representative image indicate examples of cells with cytoplasmic dilation (a, right). Quantification of GFP-positive cells with dilated cytoplasm (b, n = 3 brains for each group) and length of *D* (c, shCTL, n = 106 cells; shMAD1, n = 113 cells). (d-e) Multipolar-to-bipolar transition analysis. Mouse embryos were electroporated with shCTL or shMAD1 at E14.5 and analyzed at E16.5. Representative images of each group (d). Categorization of bipolar or multipolar cells (e, left) and quantification of cell groups (e, right, n = 3 brains for each group). Scale bars represent 50 μm (a) and 100 μm (d). Statistical significance was assessed by Student's *t*-test (b, c, and e). Analysis with nested model was conducted (c; Supplementary Dataset). Data are presented as means ± SEM. Statistical significance: *$p<0.05$, **$p<0.01$, ***$p<0.001$, ****$p<0.0001$ or n.s. (not significant).

Supplementary Figure 5.

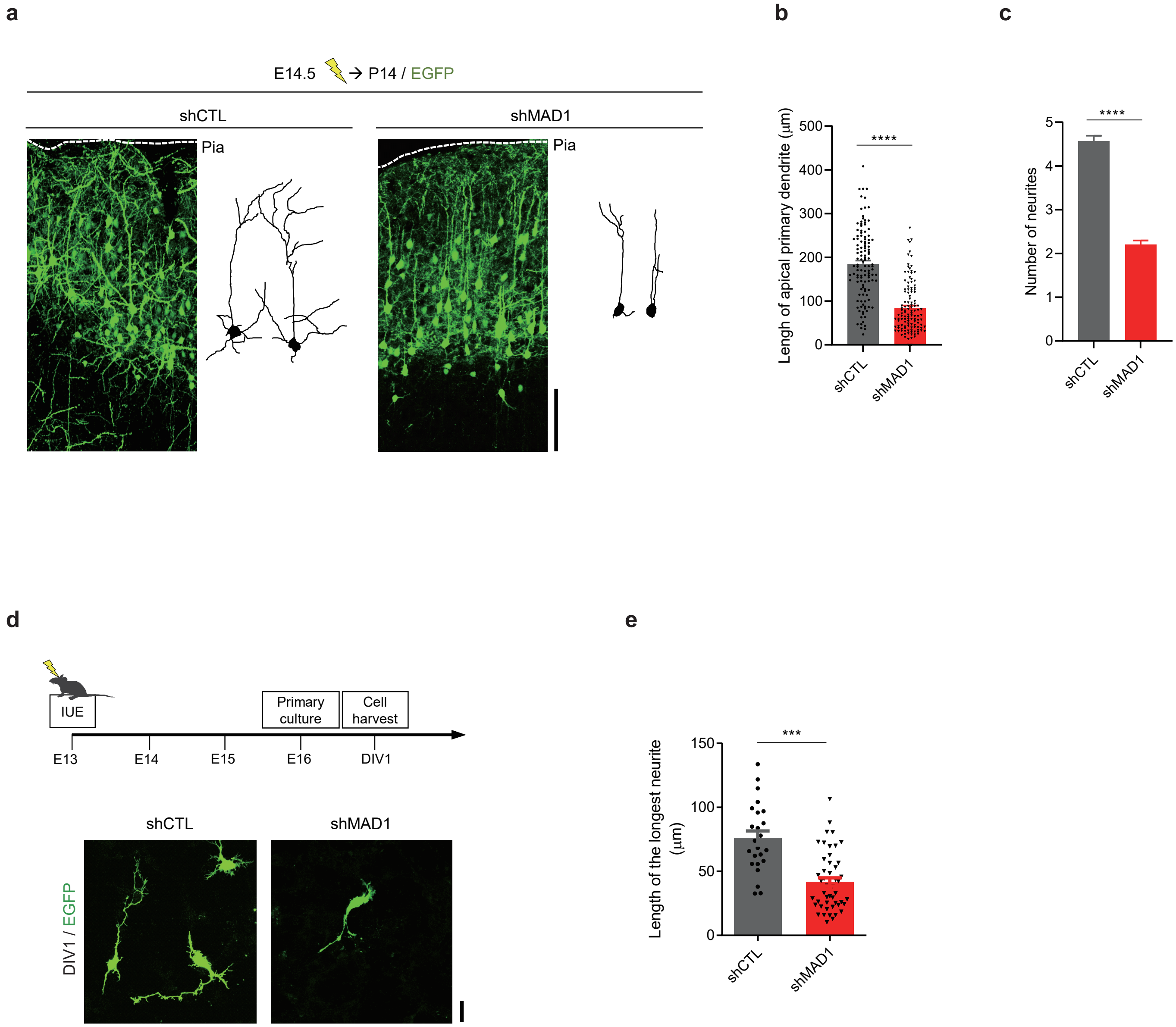

Supplementary Figure 5. Effects of embryonic MAD1-deficiency on neurite outgrowth in extended postnatal stages

(a-c) Mouse embryos were electroporated *in utero* with shCTL or shMAD1 at E14.5 and analyzed at P14. Representative images (a), quantifications of the apical dendrite length (b), and number of neurites (c, shCTL, n = 121 cells from 3 brains; shMAD1, n = 123 cells from 3 brains). (d-e) Mouse embryos were electroporated *in utero* at E13.5 with shCTL or shMAD1. E16.5 cortices were harvested and subjected to primary neuron culture for the analysis at DIV1. Experimental scheme (d, upper) and representative images (d, lower). Quantification of the longest neurite length (e, shCTL, n = 24; shMAD1, n = 49). Scale bars represent 100 μm (a) and 10 μm (d). Statistical significance was assessed by Student's *t*-test (b, c, and e). Analysis with nested model was conducted (b, c and e; Supplementary Dataset). Data are presented as means ± SEM. Statistical significance: *$p<0.05$, **$p<0.01$, ***$p<0.001$ or ****$p<0.0001$.

Supplementary Figure 6.

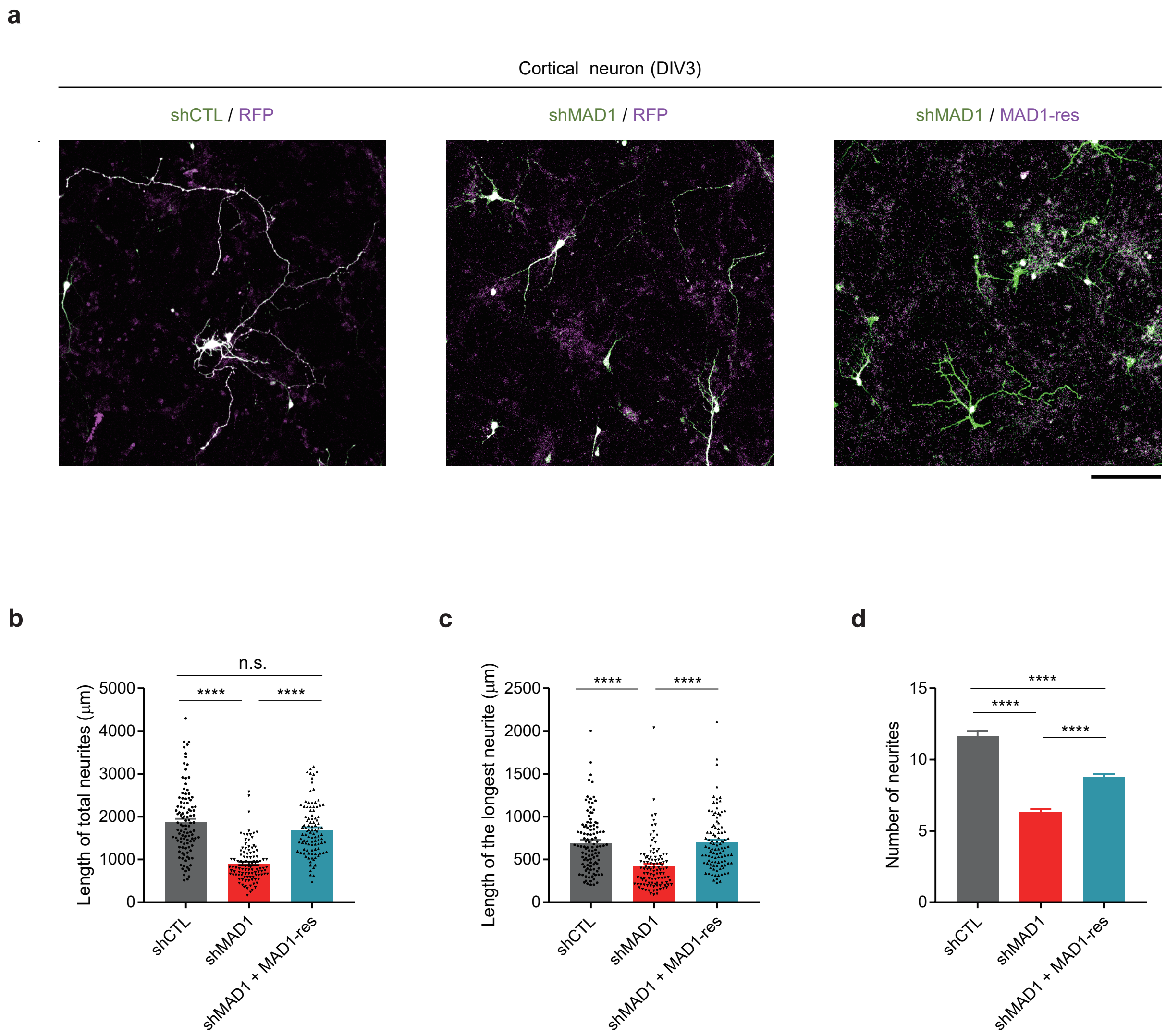

Supplementary Figure 6. Reversal of MAD1-deficiency phenotype by an shRNA-resistant form of MAD1

(a-d) Mouse cortical neurons were transfected with constructs as indicated at DIV1 and fixed at DIV3 for imaging. Representative images of each group (a). Length of total neurites (b), length of the longest neurite (c), and number of neurites (d, shCTL, n = 109; shMAD1, n = 106; shMAD1+MAD1-res, n = 102) were analyzed. Scale bar represents 100 μm (a). Analysis with nested model was conducted (b-d; Supplementary Dataset). Data are presented as means ± SEM. Statistical significance was determined by one-way ANOVA followed by Turkey's post-hoc test: ****p<0.0001 or n.s. (not significant).

Supplementary Figure 7.

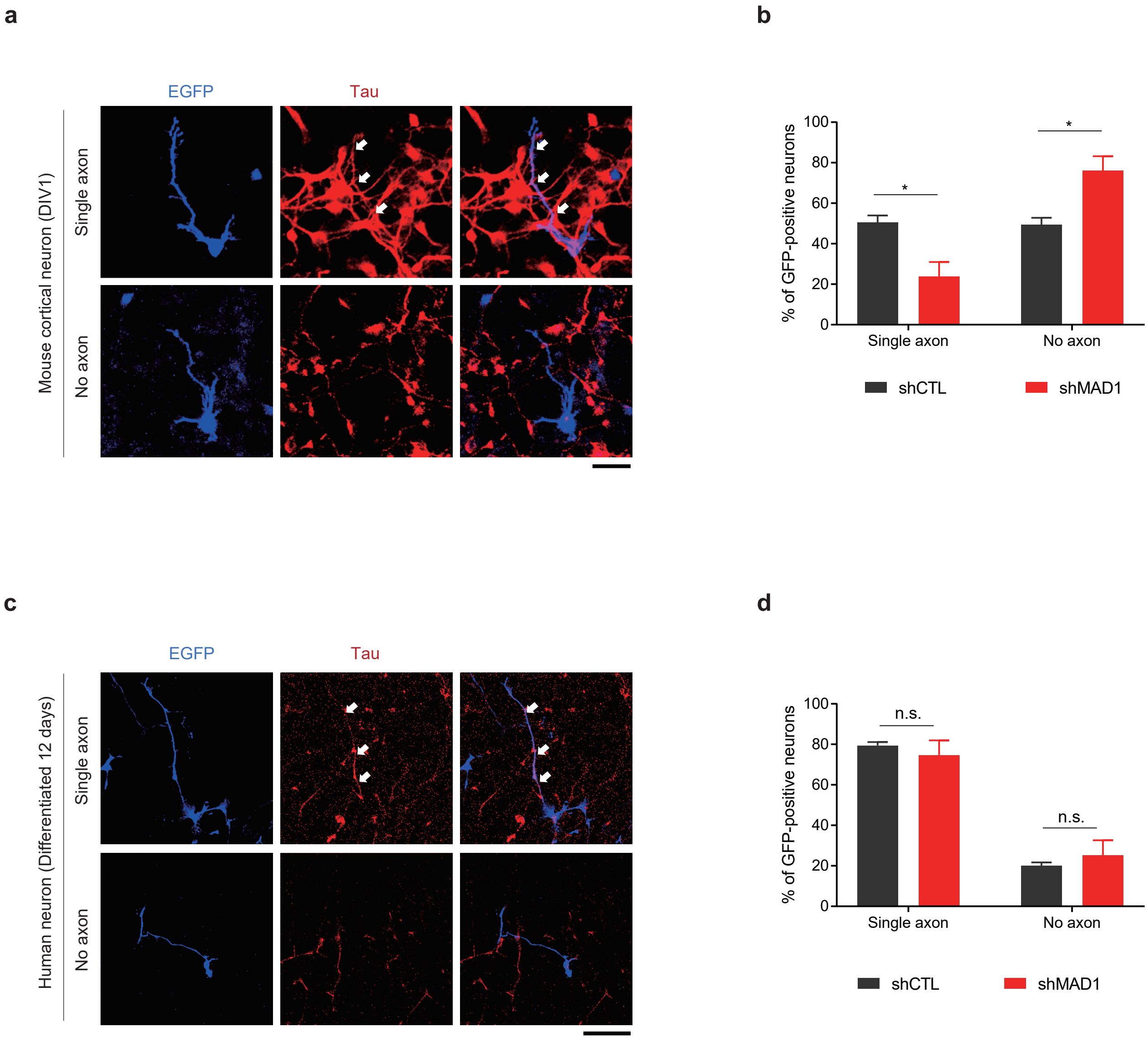

Supplementary Figure 7. Effect of MAD1-deficiency on axonal differentiation

(a-b) Mouse cortical neuron analysis. Mouse embryos were electroporated with shCTL or shMAD1 constructs at E13.5, and E16.5 cortices were applied to the primary neuron culture. Cultured neurons were fixed at DIV1 and immunostained for Tau, an axonal marker. (a) Representative images of ‘single axon’ or ‘no axon’ group (a) and quantitation (b, N = 3 for each group, n > 30 cells for each N). (c-d) Human NPC analysis. Human NPC was plated and transfected with shCTL or shMAD1 on Day 1 [3]. On day 12, differentiated neurons were fixed and immunostained for Tau. Representative images of ‘single axon’ or ‘no axon’ group (c) and quantitation (d, shCTL, N = 4; shMAD1, N = 3, n > 30 cells for each N). Scale bars represent 20 μm for (a) and 100 μm for (c). Arrows indicate the axon (a and c). Data are presented as means ± SEM. Statistical significance assessed by Student’s *t*-test: *p<0.05 or n.s. (not significant).

Supplementary Figure 8.

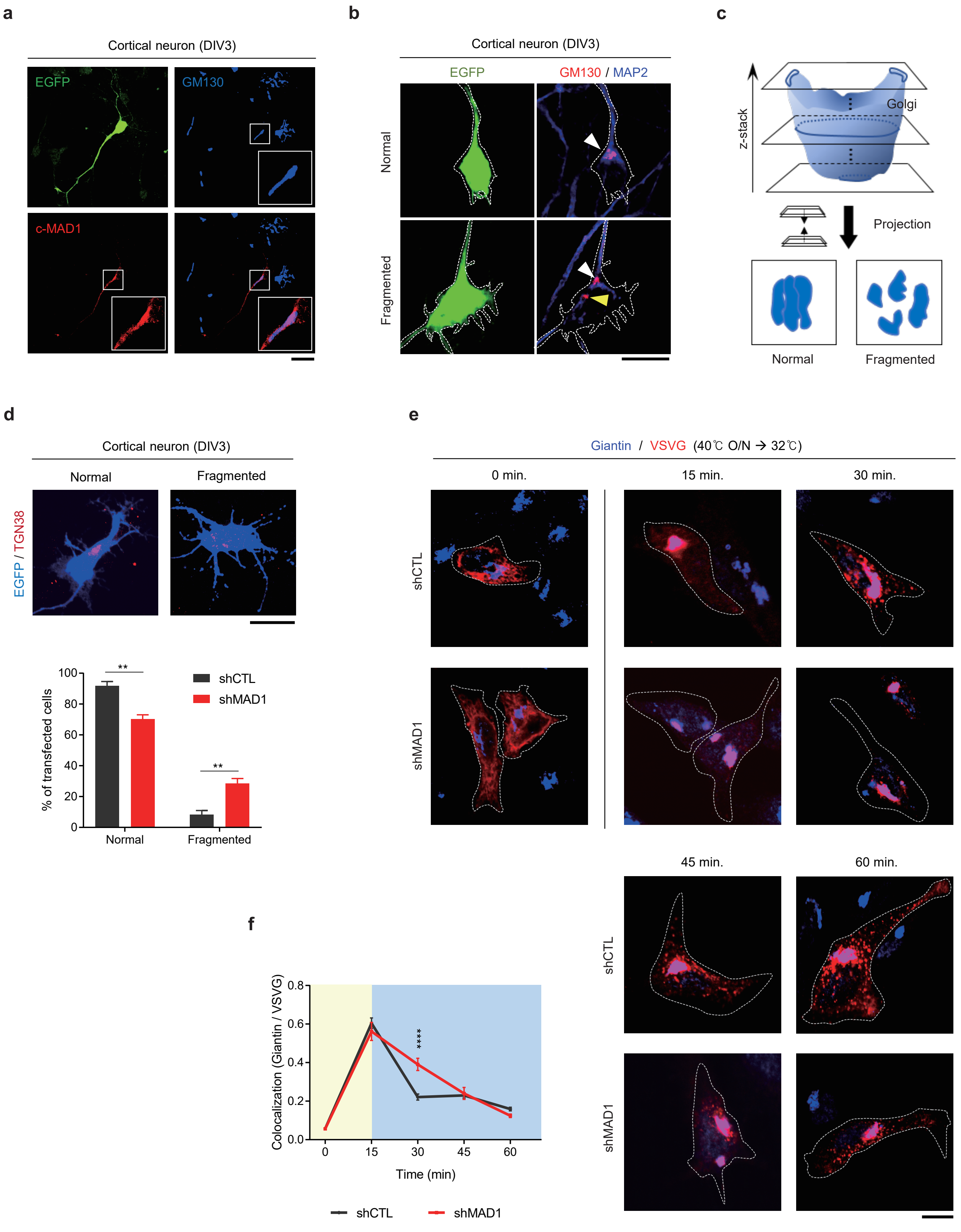

Supplementary Figure 8. Effect of MAD1 knockdown on Golgi morphology and trafficking

(a) Golgi-localization of c-MAD1. Cortical neurons were transfected with constructs for EGFP and c-MAD1 (Red). Neurons were fixed and immunostained with *cis*-Golgi marker, GM130 (blue). (b) Neuronal Golgi morphology and positioning. MAP2 (blue) was used as a dendritic marker. White arrowheads indicate normally stacked Golgi near the initiation site of the thickest dendrite. A yellow arrowhead points to fragmented Golgi deviated from the dendrite initiation site. EGFP was used as a morphology marker, and a dashed line outlined the cell surface. (c) Diagram showing the imaging process and assessment of Golgi morphology. Images were acquired via z-stacking and z-projection using confocal microscopy (See also; Supplementary Methods). (d) Golgi morphology was analyzed with *trans*-Golgi marker, TGN38 immunostaining. Representative images (upper) and statistical analysis (lower, N = 3 for shCTL; N = 3 for shMAD1; n > 30 cells for each N). (e-f) Trafficking analysis in HeLa cells. HeLa cells were transfected with shRNA and VSVG constructs as indicated. Representative time-dependent images of VSVG movements after the transition from 40℃ to 32℃. The dashed line delineates the cell surface (e).

Statistical analysis of colocalization with the human *cis*-Golgi marker Giantin and VSVG. VSVG is transported from the endoplasmic reticulum (ER) to Golgi in 15 min (light-yellow background, f) and exits the Golgi after 15 min (light-blue background, f, shCTL, 0 min, n = 28; 15 min, n = 29; 30 min, n = 25; 45 min, n = 28; 60 min, n = 23; shMAD1, 0 min, n = 30; 15 min, n = 21; 30 min, n = 20; 45 min, n = 27, 60 min, n = 31 cells). Statistical significance was assessed at 30 min time point. Scale bars represent 10 μm (a, b, d, and e). Analysis with nested model was conducted (f; Supplementary Dataset). Data are presented as means ± SEM. Statistical significance assessed by Student's *t*-test: *$p<0.05$, **$p<0.01$, ***$p<0.001$ or ****$p<0.0001$.

Supplementary Figure 9.

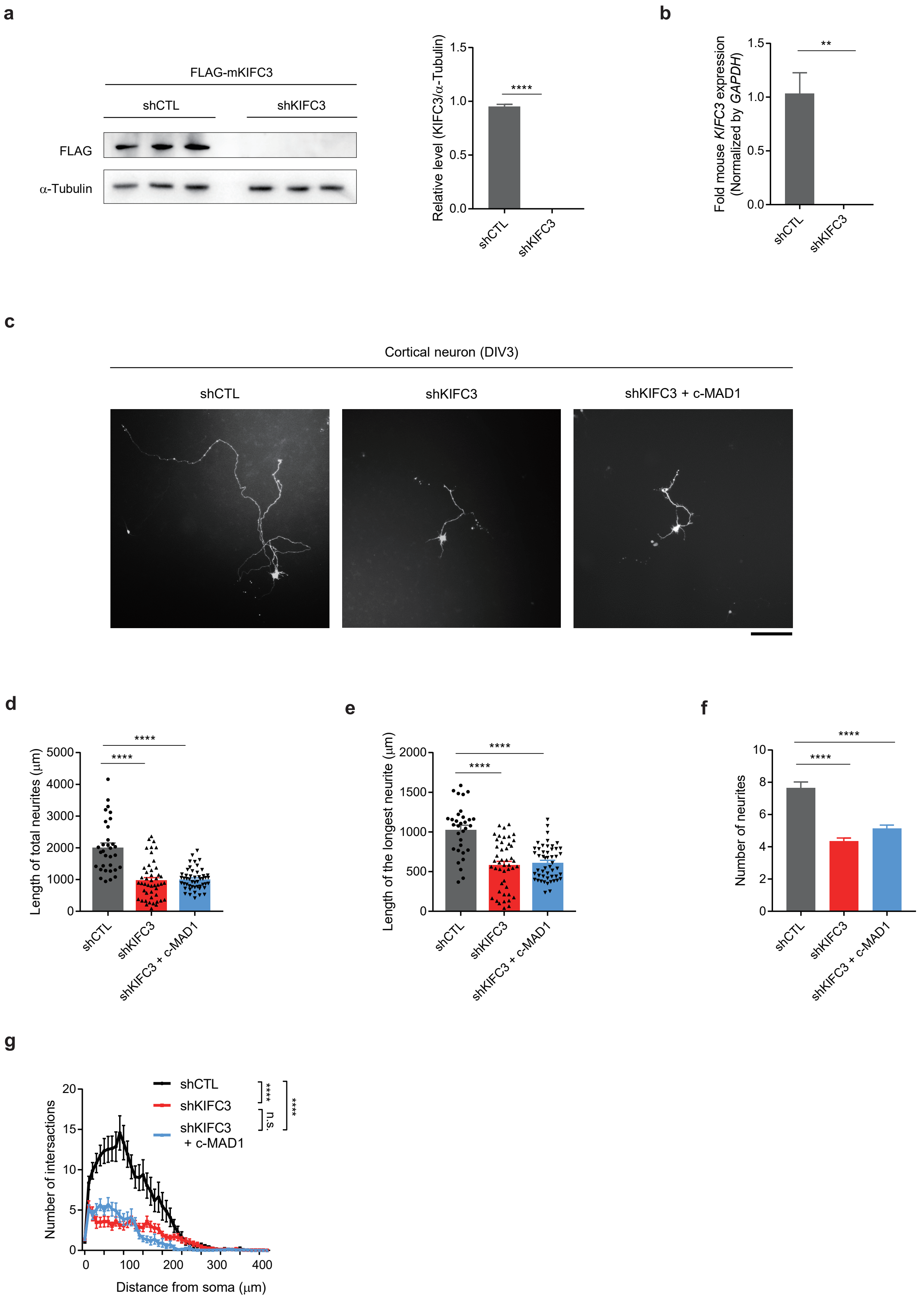

Supplementary Figure 9. Regulation of the neurite outgrowth by MAD1 and KIFC3

(a) Western blot result showing the efficiency of mouse KIFC3 (mKIFC3) shRNA (left) and quantification result (right). (b) Real-time PCR result showing the efficiency of mKIFC3 shRNA (n = 3 for each group). (c-g) Mouse neurons were transfected at DIV1 and analyzed at DIV3. Representative images from each group (c) and statistical analysis of total neurite length (d), longest neurite length (e), and number (f) of neurite outgrowth (shCTL, n = 32; shKIFC3, n = 46; shKIFC3 + c-MAD1, n = 50). Sholl analysis of neurite outgrowth (g, n = 30 for each group). Scale bar represents 50 μm (c). HEK293 cells (a) and Neuro-2a cells (b) were used. Student's *t*-test was used for statistical significance (a and b). One-way ANOVA (d-f) and two-way ANOVA (g) followed by Turkey's post-hoc test were used. Analysis with nested model was conducted (d-f; Supplementary Dataset). Data are presented as means ± SEM. Statistical significance: *p<0.05, **p<0.01, ***p<0.001, ****p<0.0001 or n.s. (not significant).

Supplementary Figure 10.

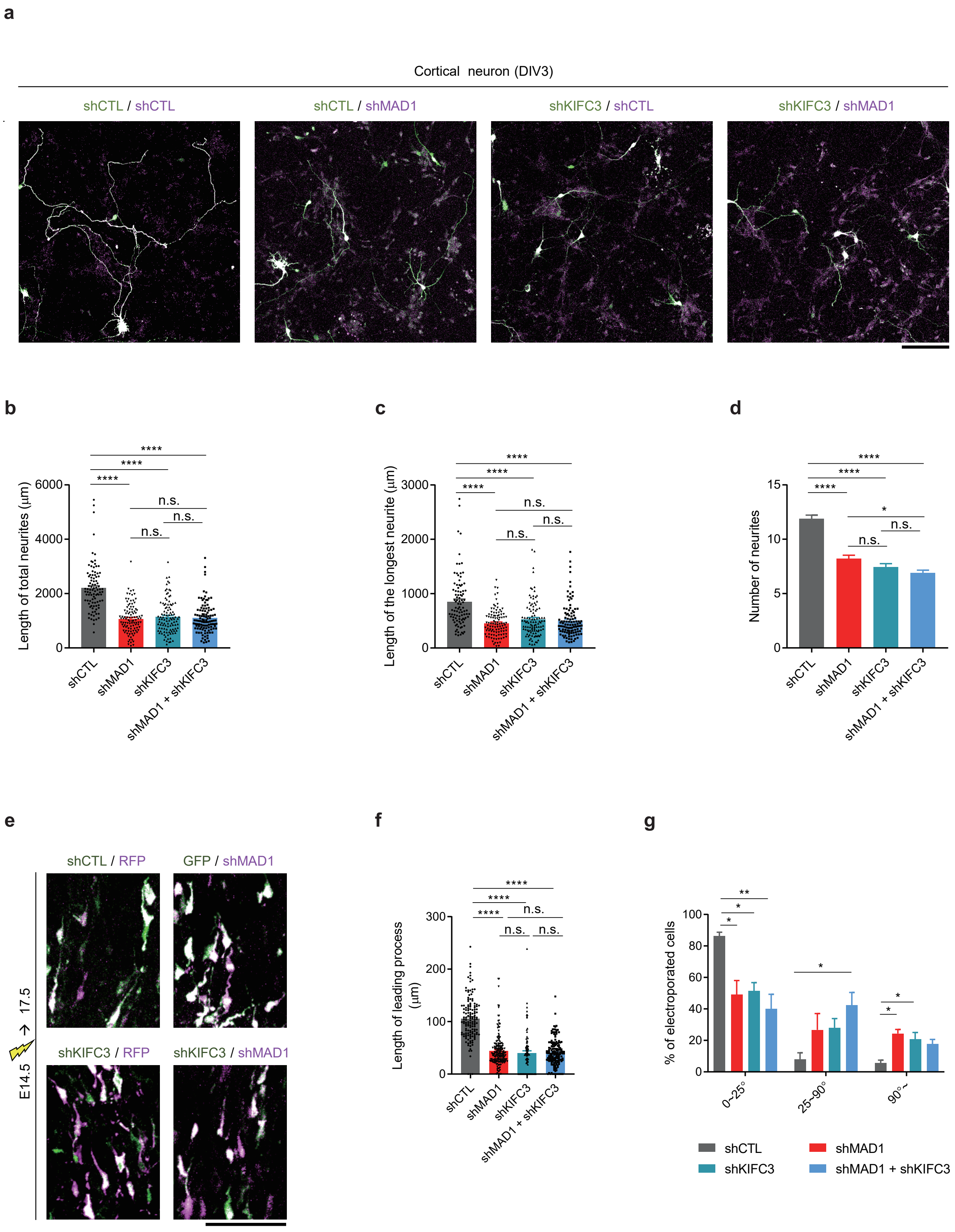

Supplementary Figure 10. Analysis of neurite outgrowth and leading process morphology upon double knockdown of MAD1 and KIFC3

(a-d) Analysis with primary neurons. Cortical neurons were transfected with shCTL, shMAD1, shKIFC3, or shMAD1+shKIFC3. Representative images of each group (a). Length of total neurites (b), length of the longest neurite (c), and number of neurites (d, shCTL, n = 95; shMAD1, n = 103; shKIFC3, n = 105; shMAD1+shKIFC3, n = 109) were analyzed. (e-g) Analysis in the developing mouse brain. Mouse embryos were electroporated at E14.5 and analyzed at E17.5. Representative images of each group (e). Length of leading process (f, shCTL, n = 140; shMAD1, n = 178; shKIFC3, n = 85; shMAD1+shKIFC3, n = 163) and directionality (g, n = 3 brains for each group). Scale bars represent 100 μm (a) and 50 μm (e). Analysis with nested model was conducted (b-d and f; Supplementary Dataset). Data are presented as means ± SEM. Statistical significance assessed by one-way ANOVA followed by Turkey's post-hoc test: *$p<0.05$, **$p<0.01$, ***$p<0.001$, ****$p<0.0001$ or n.s. (not significant).

References

1. Akera, T., Y. Goto, M. Sato, M. Yamamoto,Y. Watanabe, *Mad1 promotes chromosome congression by anchoring a kinesin motor to the kinetochore.* Nature cell biology, 2015. **17**(9): p. 1124-1133.
2. Zhou, H., T. Wang, T. Zheng, J. Teng,J. Chen, *Cep57 is a Mis12-interacting kinetochore protein involved in kinetochore targeting of Mad1–Mad2.* Nature communications, 2016. **7**(1): p. 1-13.
3. Baek, S.T., B. Copeland, E.-J. Yun, S.-K. Kwon, A. Guemez-Gamboa, A.E. Schaffer *et al.*, *An AKT3-FOXG1-reelin network underlies defective migration in human focal malformations of cortical development.* Nature medicine, 2015. **21**(12): p. 1445-1454.
