# Supplementary material for: Schizophrenia-associated Mitotic Arrest Deficient-1 (MAD1) regulates the polarity of migrating neurons in the developing neocortex
Source: Mol Psychiatry. 2022 Nov 10;28(2):856–70. doi: 10.1038/s41380-022-01856-5 (PMC9908555; doi:10.1038/s41380-022-01856-5)
Supplement: Supplementary file 2 — Supplementary Methods [file 41380_2022_1856_MOESM2_ESM.pdf]

Supplementary Methods

Antibodies

Anti-MAD1 rabbit polyclonal (Cat# GTX109519, GeneTex), anti-MAD1 rabbit polyclonal (Cat# ab175245, abcam), anti-KIFC3 mouse monoclonal (Cat# sc-365494, Santa Cruz Biotechnology), anti-GM130 mouse monoclonal (Cat# 610822, BD Bioscience), anti-Giantin rabbit polyclonal (Cat# ab80864, abcam), anti-FLAG rabbit polyclonal and mouse monoclonal (Cat# F7425 and Cat# F1804, Sigma-Aldrich), anti-GFP mouse monoclonal (Cat# sc-9996, Santa Cruz Biotechnology), anti-GFP rabbit polyclonal (Cat# A-11122, Molecular Probes), anti-c-Myc mouse monoclonal (Cat# sc-40, Santa Cruz Biotechnology), anti-α-tubulin mouse monoclonal (Cat# 66031-1-Ig, Proteintech Group), anti-MAP2 mouse monoclonal (Cat# M9942, Sigma-Aldrich), anti-Tau (Cat# 13-6400, ThermoFisher Scientific), anti-Tau (Cat# ab80579, Abcam), anti-TGN38 (Cat# sc-27680, Santa Cruz Biotechnology), anti-BrdU (Cat# PA5-32256, Invitrogen), anti-SOX2 (Cat# ab5603, Merck Millipore), anti-NeuN (Cat# MAB377, Merck Millipore), anti-γ-tubulin mouse monoclonal (Cat# sc-17787, Santa Cruz Biotechnology) and rabbit polyclonal (Cat# ab11317, abcam) were used for immunofluorescence and immunoblotting.

Mouse antibody against VSVG (BW8G65) was used as previously described [1, 2]. Normal mouse IgG (Cat# sc-2025, Santa Cruz Biotechnology) was used as a negative control for immunoprecipitation. HRP-conjugated sheep anti-mouse IgG (Cat# NA931, GE Healthcare) and donkey anti-rabbit IgG (Cat# NA934, GE Healthcare) were used as secondary antibodies for immunoblotting. Alexa Fluor 405, Alexa Fluor 488, Alexa Fluor 568, and Alexa Fluor 647 conjugated goat anti-mouse IgG (Cat# A-31553, Cat# A-11001, Cat# A-11004, and Cat# A-21236, Molecular Probes) and anti-rabbit IgG (Cat# A-31556, Cat# A-11008, Cat# A-11036, and Cat# A-21244, Molecular Probes) were used as secondary antibodies for immunocytochemistry.

Plasmids

pFLAG-CMV2 (Sigma-Aldrich), pEGFP-C3 (Clontech), pEGFP-N1 (Clontech), pmRFP-N1 (Clontech), pIRES2-DsRed2 (Clontech), and pcDNA3.1/myc-his (Invitrogen) were used for human MAD1, mouse MAD1, and mouse KIFC3 cloning. For yeast-two-hybrid screening, pPC97 and pPC86 (Invitrogen) vectors were used. FLAG-tagged c-MAD1 cloned in pCIG2-mRFP was used for *in utero* electroporation. Human and mouse *MAD1L1* cDNA clones (NCBI Reference Sequence; NM_003550.3 and NM_010752.3, respectively) were purchased from Korea Human Gene Bank, Medical Genomics Research Center (KRIBB). Mouse KIFC3 cDNA was amplified by PCR and cloned into pFLAG-CMV and mRFP-N1 vectors. All shRNA constructs were cloned into pLentiLox (pLL) 3.7 vector as previously described [3]. Oligonucleotide sequences for the mouse shMAD1 were shRNA #1; 5'-CAACTTCATTTCTCAGCGAAT-3' and shRNA #2; 5'-AGCTGAATCTAGCTTCTCATT-3'. Human MAD1 shRNA targeting sequences were 5'-CCAAAGTGCTGCACATGAG-3' for shRNA #1 [4] and 5'-CAGGCAGTGTCAGCAGAAC-3' for shRNA #2 [5], respectively. In mouse experiments, shRNA#1 was used. Mouse KIFC3 shRNA targeting sequence was 5'-GGCAGTACACGAGAACCTG-3'. The targeting site of mouse MAD1 shRNA#1 was changed to 5'-TAATTTTATCTCGCAACGGAT-3' by site-directed mutagenesis to make it an shRNA-resistant form (MAD1-res).

RT-PCR and quantitative real-time PCR

For semi-quantitative RT-PCR, C57BL/6 mice brains (E10, E12, E14, E16, E18, P1, and adults) were isolated, and total RNAs were extracted with TRI-Solution Kit™ (Bio Science Technology) following the manufacturer's instructions. cDNA was synthesized with ImProm-II™ Reverse Transcription System (A3800) by manufacturer's guide from 1 μg RNA of each group. Equal amounts of synthesized cDNAs were used for quantitative comparison. *GAPDH* (*Glyceraldehyde-3-phosphate dehydrogenase*) was used as an internal control. The forward and reverse primer sequences for semi-quantitative RT-PCR were 5'-CTGCGGGAACAGGAGGACAG-3' and 5'-ACTGCAGCTCCGAGACACGC-3' for mouse

MAD1L1.

Quantitative real-time PCR (qRT-PCR) was performed using the FastStart Universal SYBR Green Master (Roche) and the StepOnePlus thermocycler (Applied Biosystems). The relative expression among the groups was calculated using the $2^{-\Delta\Delta Ct}$ method. The forward and reverse primer sequences, respectively, were 5'-CACATGAGCCTTAACCCAATCA-3' and 5'-CGGTCATGGTCCTCATGCT-3' for mouse *MAD1L1*, 5'-CCTCATTACCTCCTGCATCGA-3' and 5'-TCTTGCCGGCACCTGTCT-3' for mouse *KIFC3*, 5'-CACTGAAGGGCATCTTGG-3' and 5'-TTACTCCTTGGAGGCCATG-3' for mouse *GAPDH*. 5'-CTGCGTGAGAAAGAGGACAGTCT-3' and 5'-TTCCGAGATCCTCCCTTCAGT-3' for human *MAD1L1*, and 5'-GGGCATCCTGGGCTACACT-3' and 5'-TTACTCCTTGGAGGCCATG-3' for human *β-actin*.

Cell line and primary culture of neurons

HEK293 and HeLa cells were cultured with DMEM (Welgene) supplemented with 10% (v/v) FBS (Fetal Bovine Serum, Gibco) and 1% Antibiotic-Antimycotic (Gibco). Cell lines were authenticated by the STR profiling method. Cell lines were tested as negative for mycoplasma contamination.

For primary neuron cultures, pregnant mice were purchased from Hyochang Science (Daegu, Korea). Pregnant mice were sacrificed, and E15~E16 embryos were used for culture. Isolated cortical or hippocampal tissues were kept in HBSS (Gibco) on ice. Tissues were dissected with 0.25% trypsin (Sigma-Aldrich) and 0.1% DNase I (Sigma-Aldrich) addition and incubated for 10 min at 37℃. Cells were resuspended in plating media prepared with DMEM supplemented with 10% (v/v) FBS. The final cell concentration was 4.0 x 10^5 cells/mL and plated on glass coverslips coated with poly-D-lysine and laminin. 2 hr post-plating, the medium was changed to neurobasal medium containing 2 mM glutamine, 2% (v/v) B27 supplement (Gibco), and 1% (v/v) penicillin/streptomycin.

Transfection

HEK293 and HeLa cells were transfected with VivaMagic (Vivagen) or polyethyleneimine (PEI) solution (1 mg/mL stock) by following the manufacturer's instructions. Neuro-2a cells and neurons were transfected with Lipofectamine2000 (Invitrogen) at least 12 hr after plating. Cell confluencies (60~80%) were adjusted according to the experimental purposes. After 18~22 hr of cell plating, cell conditions and confluencies were monitored by light microscopy. Amounts of total DNA (μg / well) were varied by culture sizes (500 μg in 24-well plates, 1 μg in 12-well plates, 2 μg in 6-well plates, and 10 μg in 10cm culture dish). 1 μg of DNA and 3 μL of transfection reagents were mixed with Opti-MEM (Gibco). The mixtures were incubated for 15 min at RT. The medium was replaced with a culture medium 3 hr after transfection.

Immunoblotting and immunoprecipitation

Mouse brain tissues or transfected cells were washed with 1X PBS and lysed with 1X erythrocyte lysis buffer (50 mM Tris, pH 8.0, 250 mM NaCl, 0.1% NP-40, 5 mM EDTA, 2mM sodium pyrophosphate, 5 mM NaF, 2 mM Na_3VO_4, 1mM DTT, and protease inhibitor cocktail (Roche)). Sonicator was used for complete lysis. For immunoprecipitation, 1~2 μg of antibody was added to the lysate and incubated at 4℃ overnight on a rotator. 20 μL of Protein-A agarose beads (Roche) was added and incubated for 2~3 hr at 4℃ on a rotator. The mixtures were washed 3 to 5 times with lysis buffer. For immunoblotting, 5X SDS sampling buffer (2% SDS, 60 mM Tris pH 6.8, 24% glycerol, 0.1% bromophenol blue, 5% β-mercaptoethanol) was added, and the mixtures were boiled at 100℃ for 10 min. SDS-PAGE was performed with 8 or 10% polyacrylamide gel in an electrophoresis tank (Bio-Rad) and transferred to PVDF membrane (Millipore). Transferred membranes were blocked with 5% skim milk in Tris-buffered saline (20 mM Tris pH 8.0 and 137.5 mM NaCl) with 0.25% Tween20 (TBST) for 0.5~1 hr at room temperature (RT). The primary antibody was mixed with skim milk followed by the manufacturer's guide,

and membranes were incubated at 4℃ overnight. The membranes were further incubated with HRP-conjugated secondary antibodies for 1 hr at RT, and protein signals were detected by ECL solutions (Bio-Rad) in the image analyzer (Azure Biosystems).

Immunocytochemistry and immunohistochemistry

For immunocytochemistry (ICC), transfected cells were washed with PBS and fixed with 4% paraformaldehyde and 4% sucrose in PBS for 5~10 min. The additional fixation with -20℃ methanol for 20 min was followed if needed. Cells were permeabilized with 0.2% Triton X-100 in PBS for 10 min and incubated in the blocking solution (4% BSA in PBS) for 1 hr at RT. Cells were incubated with primary antibodies at 4℃ overnight and washed with PBS for 10 min, three times. Incubation with secondary antibodies (Alexa Fluor 405-, Alexa Fluor 488-, Alexa Fluor 568-, and Alexa Fluor 647-conjugated) for 1 hr at RT were followed depending on each experimental purpose. Finally, coverslips were placed on slide glasses and fixed with mounting media (Biomeda).

For immunohistochemistry (IHC), harvested brains were washed with PBS and fixed with 4% paraformaldehyde and 4% sucrose in PBS overnight, followed by sequential changes of sucrose concentration, 10% in PBS for one day, 20% in PBS for one day, and 30% in PBS for one day. Brains were frozen with OCT solution (Leica Biosystems) and sectioned using cryostats (Leica Biosystems) with 10~50 μm thickness depending on the experimental purpose. Then, dissected tissue was fixed to Superfrost Plus microscope slides (Thermo Fisher Scientific) and dried at least for 6 hr at RT. Dissected tissues were examined with fluorescent microscopy to confirm the electroporated cells with fluorescence. Dried samples were washed with PBS and permeabilized with 0.2% Triton X-100 in PBS for 10 min. The antigen retrieval process was followed at 95℃, 10 min with citrate buffer of pH 6.0. Blocking was conducted with CAS-Block histochemical reagent (Thermo Fisher Scientific) for 1 hr at

RT. Samples were incubated with primary antibody mixtures at 4℃ overnight and incubated with secondary antibody mixtures for 1~2 hr at RT. For nuclear staining, samples were incubated with Hoechst solution. Finally, coverslips were placed on slide glasses and fixed with mounting media (Biomeda). Images of brain tissue samples were acquired using confocal microscopy with z-stacks of 0.5~1 μm intervals after confirming the electroporated regions by fluorescence signals. Each z-stack image and a merged z-projection image were compared to discriminate the cell periphery.

In utero electroporation

Plasmids for injection were isolated by EndoFree plasmid maxi kit (QIAGEN). DNA solution (≥1.5 μg/μL) was mixed with 0.001% Fast Green solution (Sigma-Aldrich) and injected with pulled microcapillary tube (Drummond Scientific). Pregnant C57BL/6 mice were anesthetized with isoflurane (induction in a chamber: 2.8%, surgery via mask: 2.5%). Electroporation was conducted with an electroporator (Harvard Apparatus) with tweezer-type electrodes by 45V, 50 ms, 6 times with 531 ms intervals. After the incision was sutured, mice were kept in their home cage. Pregnant E14.5 mice were injected, and brain tissues were harvested at E17.5, E18.5, P7, P14, or P21 to analyze neuronal migration and differentiation. To analyze cell proliferation or neuronal differentiation, E13.5 embryos were electroporated *in utero*, and brain tissues were harvested at E15.5. BrdU (Sigma-Aldrich) was administered at E14.5 by intraperitoneal injection (50mg/kg in PBS). To analyze multipolar-to-bipolar transition, E14.5 embryos were electroporated *in utero*, and brain tissues were harvested at E16.5. To analyze initial neurite outgrowth and axonal determination, E13.5 embryos were electroporated *in utero*, and brain tissues were harvested at E16.5 for primary neuron culture.

Images of brain samples were acquired using confocal microscopy with z-stacks of 0.5~1 μm intervals after examining the electroporated regions by fluorescence signals. Only transfected neurons marked by fluorescence were analyzed. Each z-stack image and a merged z-projection image were compared

to discriminate the cell periphery. To minimize the sample-by-sample variation, we analyzed the samples in the comparable tissue quality, transfected region, and total transfection efficiency in each experimental set.

Neurite tracing and Sholl analysis

Primary neurons were transfected with shCTL or shRNA cloned into the pLL3.7 vector to induce knockdown. All shRNAs, including scrambled shRNA (shCTL), were cloned into the pLL3.7-EGFP or pLL3.7-mRFP vector, enabling discrimination of transfection and cell morphology by fluorescence. c-MAD1, MAD1-res (an shRNA-resistant form of full-length mouse MAD1), or KIFC3 constructs cloned in pmRFP-N1 were used for overexpression. Cells were fixed with paraformaldehyde 3 days after transfection. EGFP-positive neurons for shCTL and shRNA groups and EGFP/mRFP-positive neurons for c-MAD1, MAD1-res, or KIFC3 co-transfected groups were analyzed. To measure the neurite length of c-MAD1 or KIFC3 single overexpression, the control EGFP vector was co-transfected for a morphological marker. Images were acquired using 20x (0.45 N.A.) or 40x (0.60 N.A.) objective lens (Olympus). The length and number of neurites were measured by tracing EGFP-positive signals with Image J software (NCBI). For Sholl analysis, each neuron was traced by 10 μm radius step size with the Sholl Analysis plug-in of Image J software. All experiments were independently repeated at least three times.

VSVG Trafficking and Golgi morphology analysis

The temperature-sensitive mutant version of vesicular stomatitis virus G protein (VSVG) was cloned into pcDNA3.1-myc/His [6]. HeLa cells were transfected with shCTL or MAD1 shRNA on the first day. On the second day, the VSVG construct was transfected. After 24 hr, cells were incubated at a non-

permissive temperature (40℃) overnight to induce accumulation of unfolded VSVG proteins in ER. Cells were incubated at a permissive temperature (32℃) to induce sequential membrane trafficking from ER to Golgi and Golgi to plasma membrane.

For the analysis of post-Golgi membrane trafficking in neurons or HeLa cells, cells were incubated at 40℃ overnight after 12 hr of VSVG transfection. Cells were incubated at 20℃ for 3 hr to induce Golgi accumulation of VSVG proteins, followed by incubation at 32℃ to induce trafficking from Golgi to the plasma membrane. Cells were fixed with 4% paraformaldehyde for 10 min and blocked with PBS supplemented with 10% FBS and 0.04% sodium azide for 1 hr or overnight at 4℃. For immunostaining, antibody solution was incubated in PBS mixed with 10% FBS, 0.2% saponin, and 0.04% sodium azide. As Golgi markers, α-Giantin was used for HeLa cells, and α-GM130 was used for mouse neurons. Imaging was conducted using confocal microscopy (Olympus FV3000). The acquired image was analyzed using CellSens software (Olympus) to measure Mander's overlap coefficient.

For the analysis of Golgi morphology, neurons were transfected as in the neurite outgrowth assay. All shRNAs, including scrambled shRNA (shCTL), were cloned into pLL3.7-EGFP or pLL3.7-mRFP vector to discriminate transfection and cell morphology by fluorescence. On DIV3, transfection efficiency and cell condition were assessed. Cells were immunostained with human *cis*-Golgi marker Giantin, mouse *cis*-Golgi marker GM130, or mouse *trans*-Golgi marker TGN38. Images were acquired by *z*-stack from top to bottom of each cell with a 60x or 100x lens confocal microscopy. Cells were discriminated by comparing individual stack images with z-projection images. Transfected cells were discriminated by EGFP-positive signal and chosen for the imaging.

Cells were classified by morphological traits and position of Golgi traced by the Golgi marker. To exclude small vesicles nonspecifically labeled, significantly large size particles with > 2μm diameter were counted. Images were compared in the same intensity, brightness, and contrast. Golgi stacks with a continuous structure were classified as a "normal" group, and Golgi complexes dispersed as particles

are classified as a "fragmented" group. In neurons, Golgi stacks are classified as "dendritic" or "non-dendritic" by positioning of major Golgi fractions on the nucleus-dendrite axis. All the experiments for trafficking and Golgi morphology analyses in neurons (shCTL, shMAD1, shKIFC3, c-MAD1, shMAD1 + c-MAD1, and shKIFC3 + c-MAD1) were done in the same set for the multiple direct comparisons. All experiments were repeated at least three times.

Image analysis and preparation

Neuronal morphology was assessed with a morphological marker (EGFP) by saturating the soma region and z-projection. Brightness and contrast were adjusted in the neuronal representative images to show the intracellular signals and neuronal morphology simultaneously. Regarding IUE representative images, EGFP intensity was adjusted to show the clear morphology of each neuron, avoiding excessive saturation in soma regions to minimize difficulties in discriminating individual cells.

Images were obtained by using FV3000 confocal laser scanning microscope (Olympus) with 10x (0.4 N.A.), 20x (0.75 N.A.), 40x (0.95 N.A.), 60x (1.35 N.A.), or 100x (1.4 N.A.) objective lens. Due to the limitation of the confocal microscopy resolutions (≥230nm for 100x and ≥240nm for 60x), brightness and contrast were adjusted for the clarity of the images. Color scales were also carefully adjusted to avoid the potential overlap of the red and green signals.

Acquired images were analyzed with Image J software (NCBI) for evaluating neurite outgrowth, neuronal migration, or densitometric quantification of western blot results. CellSens software (Olympus) was used for analyzing colocalization.

Yeast-two-hybrid screening

For bait construct, human MAD1 (hMAD1) was cloned into pPC97 vector containing the DNA-binding domain of GAL4 and *Leu* gene. Human fetal brain cDNA library in pPC86 vector (GibcoBRL) containing GAL4 activation domain and *Trp* gene was used as prey. MaV203 yeast strain was used for screening, and transformation was conducted as previously described [7]. Transformed yeasts were incubated at 30℃ for 3 days. A total 3,375,000 co-transformants grown on *Leu- / Trp-* medium was initially screened with growth test on synthetic defined (SD) medium (*Leu-*, *Trp-*, *His-*, and *Ura-* containing 20 mM of 3-amino-1,2,4-triazole (3-AT; Sigma-Aldrich)). 46 putative positive colonies were transferred to SD *Leu-, Trp-* medium for β-galactosidase activity test with X-gal (Sigma-Aldrich) solution. Finally, 15 positive colonies were selected. Prey plasmids were isolated from positive colonies by incubating on *Trp-* media and prepared with Lyticase (Sigma-Aldrich). Plasmids were amplified by transformation into *DH5α* and analyzed by DNA sequencing and database (NCBI BLAST).

Human neural progenitor cell culture and neural differentiation

Human neural progenitor cells (hNPCs) were generated from H9 human embryonic stem cells (hESCs, WiCell, WA09), following the monolayer differentiation protocol of StemDiff SMADi Neural induction Kit (Stem Cell Technologies). After 3 passages, hNPCs were plated onto Matrigel (Corning) coated plate and cultured with NBF media (DMEM-F/12 supplement with 0.5X B27 (Gibco), 0.5X N2 (Gibco), 100X penicillin-streptomycin (Gibco), and 20ng/ml bFGF (Stem Cell Technologies)). The passage number of the hNPC was kept within 11 to 14 to ensure similar cellular activity. One day before transfection of shRNA into hNPCs, cells were dissociated with Accutase (Sigma-Aldrich) and plated onto Matrigel-coated 6-well plates. After 24 hr incubation, cells were approximately 70-80% confluent, and Lipofectamine 3000 (Invitrogen) was used for the shRNA transfection of hNPCs according to the manufacturer's protocol. 24hr after transfection, cells were cultured in fresh NB (DMEM-F/12

supplement with 0.5X B27, 0.5X N2, and 100X penicillin-streptomycin) media for neural differentiation. The medium was partially replaced every second day. On day 12 of differentiation, hNPCs were fixed with 4% PFA (Fisher Chemical) for 10 min at room temperature. All cell lines were mycoplasma-negative in the routine PCR-based mycoplasma detection tests.

Forebrain organoid culture and electroporation

Brain region-specific cerebral organoid culture was prepared following the previous report [8]. We maintained hESC (WA09, WiCell, USA) by MEF-dependent culture with stem cell culture media (DMEM/F12 (Gibco), 20% Knockout serum replacement (KOSR, Gibco), 1x GlutaMax (Gibco), 1x non-essential amino acid solution (NEAA, Gibco), 1x Pen/Str (HyClone), 1x β-mercaptoethanol (Gibco), 10 ng/ml basic FGF (Peprotech)).

To establish forebrain organoid, we detached 1~1.5 mm sized WA09 stem cell colonies by 30 min treatment of Collagenase IV (Thermo Fisher 17104019, 1mg/ml, dissolved in DMEM/F12). The duration of Collagenase IV treatment was increased up to 2 hr until colonies were detached. Detached stem cell colonies were transferred to ultra-low attachment 6-well plate (Corning) and cultured for 4 days with Forebrain first media (DMEM/F12, 20% KOSR, 1x GlutaMax, 1x NEAA, 1x β-mercaptoethanol, 1x Pen/Str, 2 μM Dorsomorphin (Stem Cell Technologies), 2 μM A-83 (Stem Cell Technologies)). On culture day 5 and 6, half of the media was replaced with Forebrain second media (DMEM/F12, 1× N2 supplement, 1× GlutaMax, 1x NEAA, 1x Pen/Str, 1 μM CHIR-99021 (Stem Cell Technologies), 1 μM SB-431542 (Stem Cell Technologies)). On culture day 7, embryonic bodies were embedded into a mixture of Matrigel (Corning) and Forebrain second media (3:2 ratio). The mixture was spread on an ultra-low attachment plate and incubated at 37℃ for 30 min to solidify. Organoids were further cultured for 7 days with Forebrain second media. On culture day 14, Matrigel was mechanically broken by 2 or 3 times of pipetting with 10 ml pipet. Organoids were further cultured with Forebrain third media

(DMEM/F12, 1× N2 supplement (Gibco), 1× B27 supplement (Gibco), 1× GlutaMAX, 1x NEAA, 1x β-mercaptoethanol, 1x Pen/Str, 2.5 μg/ml insulin (Sigma-Aldrich)) and shaking (75 rpm).

For electroporation on forebrain organoids, we utilized organoids cultured for 50 days. DNA constructs (2 μg/μl concentration) combined with Fast green were injected into empty spaces of organoid rosettes. The injection was performed with a micro-injector (PLI-100 Pico-Injector, Harvard Apparatus) set as 10 psi and 5~20 msec of injection time. After that, electroporation was performed with an electroporator (Harvard apparatus, Holliston, MA, USA) set as 80 V, 50 msec duration, 1s interval, 5 pulses.

References

1. Yang, J.-S., H. Gad, S.Y. Lee, A. Mironov, L. Zhang, G.V. Beznoussenko*, et al.*, *A role for phosphatidic acid in COPI vesicle fission yields insights into Golgi maintenance.* Nature cell biology, 2008. **10**(10): p. 1146-1153.
2. Yang, J.-S., C. Valente, R.S. Polishchuk, G. Turacchio, E. Layre, D.B. Moody*, et al.*, *COPI acts in both vesicular and tubular transport.* Nature cell biology, 2011. **13**(8): p. 996-1003.
3. Suh, B.K., S.-A. Lee, C. Park, Y. Suh, S.J. Kim, Y. Woo*, et al.*, *Schizophrenia-associated dysbindin modulates axonal mitochondrial movement in cooperation with p150 glued.* Molecular brain, 2021. **14**(1): p. 1-14.
4. Akera, T., Y. Goto, M. Sato, M. Yamamoto and Y. Watanabe, *Mad1 promotes chromosome congression by anchoring a kinesin motor to the kinetochore.* Nature cell biology, 2015. **17**(9): p. 1124-1133.
5. Zhou, H., T. Wang, T. Zheng, J. Teng and J. Chen, *Cep57 is a Mis12-interacting kinetochore protein involved in kinetochore targeting of Mad1–Mad2.* Nature communications, 2016. **7**(1): p. 1-13.
6. Bergmann, J.E., *Using temperature-sensitive mutants of VSV to study membrane protein biogenesis.* Methods in cell biology, 1989. **32**: p. 85-110.
7. Gietz, R.D. and R.H. Schiestl, *High-efficiency yeast transformation using the LiAc/SS carrier DNA/PEG method.* Nature protocols, 2007. **2**(1): p. 31-34.
8. Qian, X., F. Jacob, M.M. Song, H.N. Nguyen, H. Song and G.-l. Ming, *Generation of human brain region–specific organoids using a miniaturized spinning bioreactor.* Nature protocols, 2018. **13**(3): p. 565-580.
